# MIG-seq is an effective method for high-throughput genotyping in wheat (*Triticum* spp.)

**DOI:** 10.1093/dnares/dsac011

**Published:** 2022-04-12

**Authors:** Kazusa Nishimura, Ko Motoki, Akira Yamazaki, Rihito Takisawa, Yasuo Yasui, Takashi Kawai, Koichiro Ushijima, Ryohei Nakano, Tetsuya Nakazaki

**Affiliations:** 1 Graduate School of Agriculture, Kyoto University, Kizugawa City, Kyoto Prefecture 619-0218, Japan; 2 Faculty of Agriculture, Kindai University, Nara City, Nara Prefecture 631-8505, Japan; 3 Faculty of Agriculture, Ryukoku University, Otsu City, Shiga Prefecture 520-2194, Japan; 4 Graduate School of Environmental and Life Science, Okayama University, Okayama City, Okayama Prefecture 700-8530, Japan

**Keywords:** MIG-seq, genotyping, wheat

## Abstract

MIG-seq (Multiplexed inter-simple sequence repeats genotyping by sequencing) has been developed as a low cost genotyping technology, although the number of polymorphisms obtained is assumed to be minimal, resulting in the low application of this technique to analyses of agricultural plants. We applied MIG-seq to 12 plant species that include various crops and investigated the relationship between genome size and the number of bases that can be stably sequenced. The genome size and the number of loci, which can be sequenced by MIG-seq, are positively correlated. This is due to the linkage between genome size and the number of simple sequence repeats (SSRs) through the genome. The applicability of MIG-seq to population structure analysis, linkage mapping, and quantitative trait loci (QTL) analysis in wheat, which has a relatively large genome, was further evaluated. The results of population structure analysis for tetraploid wheat showed the differences among collection sites and subspecies, which agreed with previous findings. Additionally, in wheat biparental mapping populations, over 3,000 SNPs/indels with low deficiency were detected using MIG-seq, and the QTL analysis was able to detect recognized flowering-related genes. These results revealed the effectiveness of MIG-seq for genomic analysis of agricultural plants with large genomes, including wheat.

## 1. Introduction

Detecting genome-wide genetic polymorphisms are essential for genetic analyses, such as linkage map construction and phylogenetic tree construction. For these genetic analyses, simple sequence repeats (SSR), restriction fragment length polymorphism, and cleaved amplified polymorphic sequences markers have frequently been used. With the recent development of Next-Generation sequencing (NGS) technology, the cost of analysis for obtaining genotype data for molecular genetics has significantly reduced. In some crops, such as rice and soy, whole genome resequencing was applied for a genome-wide association study (GWAS) with over 100 accessions.^1^^,^^2^ Protocols, such as GBS (Genotyping by Sequencing) and RAD-Seq (Restriction Site-Associated DNA Sequencing),^3^^,^^4^ which are restriction enzyme-based methods, have been developed. These methods are employed in several studies for genetic analyses because they reduce genome complexity and allow genotyping data to be obtained from large numbers of samples at a low cost. Afterward, double-digest RAD-Seq (ddRAD-seq), a method using two restriction enzymes, was reported.^5^ Recently, Genotyping by Random Amplicon Sequencing-Direct (GRAS-Di) was developed as higher throughput genotyping by polymerase chain reaction (PCR) -based method,^6^ and this technique was used for GWAS and linkage mapping in several crop species.^7–10^

Furthermore, NGS is useful for obtaining genomic polymorphisms for genomic analysis in wheat. At first, the genome of tetraploid wild species (*Triticum turgidum* L. ssp. *dicoccoides*) was sequenced and assembled, followed by the genome of a bread wheat (*Triticum aestivum* L. ssp. *aestivum*) cultivar, ‘Chinese Spring’, and the durum wheat genome (*T.turgidum* L. ssp. *durum*) were also assembled.^11–13^ Recently, *de novo* assembly of several bread wheat varieties has been achieved.^14^ However, it is still unreasonable to sequence the whole genome of more than 100 varieties for performing genetic analyses, such as GWAS, because the sequencing cost is too high for wheat. This is because wheat carries a large genome through allopolyploidization and duplication of many repetitive sequences, such as retrotransposons. Therefore, applying NGS analysis technologies with ‘reduced complexity’ for wheat and its relatives is effective, and several studies have been conducted using these methods.^9^^,^^15^^,^^16^ Here, ddRAD-seq has been reported to be applied in many cases for genetic analysis of wheat, and this method is considered to be effective, but this method requires high-quality DNA, causing high cost for extraction and purification of DNAs. Therefore, the situation is such that further methodology improvement is expected.

Multiplexed inter-simple sequence repeats (ISSR) Genotyping by sequencing (MIG-seq), one of the ‘reduced complexity’ methods for building NGS libraries by PCR similar to GRAS-Di, was developed to analyze ecological studies.^17^ With MIG-seq, libraries can be constructed in two PCR reactions: in the first PCR, the ISSR regions were amplified even from low-quality DNA, while in the second PCR, an index sequence for identifying individual samples was introduced. They reported that this method applies particularly to medium-scale studies based on less than 1,000 markers in ecology, phylogeography, and conservation genetics, including rapid studies of genetic differences among individuals (clones or varieties), populations, related species, and hybrids. However, as NGS analysis for linkage map construction is required to detect high-density single-nucleotide polymorphisms (SNPs), a MIG-seq is ineffective for genetic analysis, such as linkage mapping and GWAS. Consequently, MIG-seq has not been used for genetic analysis in major agricultural plant species so far, although this method has the advantage of not requiring high-quality DNA.

We hypothesized that MIG-seq might be useful for plant species with large genome sizes, such as wheat. There is a correlation between genome size and the number of SSRs in virus and mosquito species,^18^^,^^19^ and we can expect that the number of loci that can be sequenced by MIG-seq will increase as the number of SSRs increases. Therefore, we can assume that sufficient polymorphisms can be detected in wheat with a large genome size. We conducted the following experiments to test this hypothesis. In this study, we demonstrated: (i) investigation of the relationship among the number of bases that can be sequenced stably by MIG-seq, genome size, and the number of SSRs targeted by MIG-seq using 12 plant species that include various crops with diverse genome sizes, (ii) application of MIG-seq for population structure analysis, linkage map construction, and quantitative trait loci (QTL) analysis for heading date in tetraploid wheat to evaluate the effectiveness of MIG-seq for wheat.

## 2. Materials and methods

### 2.1. Plant materials

Four Tausch’s goatgrass (*Aegilops tauschii* L.) accessions, four tetraploid and hexaploid wheat accessions each, four capsica (*Capsicum* spp.) accessions, two quinoa (*Chenopodium quinoa* L.) accessions, two strawberry (*Fragaria vesca* L.) accessions, four melon (*Cucumis melo* L.) accessions, four radish (*Raphanus sativus* L.) accessions, four cabbage (*Brassica oleracea* L.) accessions, four rose (*Rosa* spp.) accessions, four tomato (*Solanum lycopersicum* L.) accessions, and four peach (*Prunus persica* L.) accessions were used to evaluate MIG-seq performance for plant species with diverse genome size. These accessions were selected so that there would not be only varieties of the same clonal origin (e.g. bud sport mutant) within a species. Supplementary Table S1 shows the information on these cultivars or accessions.

We used diverse tetraploid wheat collection (TWC) consisting of 195 pure accessions, including six subspecies: 55 domesticated emmer wheat (*T. turgidum* L. ssp. *dicoccum*), 114 durum wheat (*T. turgidum* L. ssp. *durum*), 10 Persian wheat (*T. turgidum* L. ssp. *carthricum*), 4 Colchis emmer wheat (*T. turgidum* L. ssp. *paleocolchicum*), 7 Polish wheat (*T. turgidum* L. ssp. *polonicum*), and 5 rivet wheat (*T. turgidum* L. ssp. *turgidum*) accessions for population structure analysis using SNP/indels obtained by MIG-seq (Supplementary Table S2). We used an F_2_ tetraploid wheat population (*n* = 127) and F_6_ tetraploid wheat recombinant inbred lines (RILs) (*n* = 176) for linkage map construction and QTL analysis (Supplementary Table S3). The F_2_ population was derived from a cross between TTW41 (*T.turgidum* L. ssp. *dicoccum*: KU-3723) and TTW139 (*T.turgidum* L. ssp. *durum*: KU-3672). KU-3723 and KU-3672 are the accession numbers in NBRP KOMUGI (https://shigen.nig.ac.jp/wheat/komugi/). This F_2_ population consisted of 127 individuals, 124 of which were used in Nishimura et al.^20^ to analyze *VRN-A3* (orthologous genes of *flowering locus T* in wheat^21^) effect on heading date. F_6:7_ RILs were derived from a cross between TN26 (*T.turgidum* L. ssp. *dicoccum*) and TN28 (*T.turgidum* L. ssp. *durum*). F_7_ progeny of F_6_ RILs were used for investigating the heading dates under field conditions for QTL analysis.

### 2.2. Growth condition and heading data evaluation in wheat population

Eight F_7_ seeds per line of RILs and 127 F_2_ seeds were germinated on wet filter paper at 20°C under 24-h day length conditions on 5 November 2017 and 23 November 2017, respectively, and transplanted into 2 **×** 2 cell trays on 7 November 2017 and 26 November 2017. These plants were grown at 20°C in phytotron without artificial light, followed by transplantation into the experimental farm of the Graduate School of Agriculture, Kyoto University (34°44′02.4″N, 135°50′16.2″E) on 27 November 2017, and 7 December 2017. In each experimental plot, we planted seedlings at a spacing of 1 m between rows and 15 cm between individuals. Then, the heading dates of these segregation populations and used days from germination to heading (DGH) were recorded for QTL analysis.

### 2.3. MIG-seq library construction and NGS sequencing

Supplementary Table S1 shows the methods for extracting DNAs^22^ for each accession of the 12 species. DNAs of TWC, the F_2_ population and F_6:7_ RILs were extracted from leaves of individuals using Dneasy Plant mini kit (Qiagen, USA), a method reported in Zheng et al.,^23^ and Nucleon PhytoPure DNA extraction kit (GE Healthcare, Buckinghamshire, UK), respectively.

To evaluate the performance of MIG-seq, in the case of the experiment of first PCR of MIG-seq for the 12 species, the concentration of template DNAs to 20-ng/μl was adjusted using NanoDrop One (Thermo Fisher, Waltham, USA). Using 16 multiplexing primers developed by Suyama and Matsuki^17^ (Supplementary Table S4) and Multiplex PCR Assay Kit ver.2 (TAKARA Bio Co. Ltd., Japan), the first PCR was performed using the following profile, referring to the method in Suyama et al.^24^: 94°C for 2 min, followed by 25 cycles at 94°C for 30 s, 38°C for 1 min, 72°C for 1 min, and a final extension at 72°C for 10 min. We performed the second PCR using Prime Star GXL DNA polymerase (TAKARA Bio Co. Ltd., Japan), the first PCR products diluted 50-fold, and the second PCR primers (Supplementary Table S5) with the following profile: 98°C for 30 s, followed by 20 cycles at 98°C for 10 s, 54°C for 15 s, 68°C for 30 s, and a final extension at 72°C for 10 min. Second PCR products of each sample in the same volume of liquid were pooled and purified using AMpure XP (Beckman Coulter, Inc., USA). The purified library was size-selected using SPRIselect (Beckman Coulter, Inc., USA). For removing fragments of small size (right side selection) and fragments of larger size (left side selection), the ratios of library sample to SPRIselect are 1:0.8 and 1:0.56, respectively. Of the 12 species, 151 bp paired-end reads of nine species except wheat and Tausch’s goatgrass were obtained using the Illumina HiSeq X. The MIG-seq libraries of wheat and Tausch’s goatgrass were sequenced using Illumina NovaSeq 6000, a pre-made library sequencing service (Novogene Co. Ltd, China), and obtained 150 bp paired-end reads of them.

To evaluate the relationship between the number of reads obtained and DNA concentration, the DNA of F_6_ RILs and their parents were diluted 10-fold without checking the DNA concentration, and were used for the first PCR of MIG-seq. Then, the concentration was measured using Qubit™ dsDNA HS Assay Kit (Thermo Fisher Scientific, USA) after the first PCR. For the F_2_ population of tetraploid wheat and TWC, the DNA concentration for MIG-seq library construction was not examined.

For TWC, we sequenced a dual-indexed library containing all samples (Supplementary Tables S2 and S5) using two lanes of HiSeq X, and 151 bp paired-end reads were obtained. In the case of the F_6:7_ RILs, 192 sample libraries from 176 F_6_ individuals and eight replicates of each of their parents were divided into four pooled libraries. Then, we added a unique dual index to 48 samples and pooled them into a single library (Supplementary Table S3). For these four pooled libraries, 150 bp paired-end reads were obtained using the Illumina NovaSeq 6000 pre-made library sequencing service. In the case of the F_2_ population, all second PCR products (143 sample libraries from 127 F_2_ individuals and eight replicates of each of their parents) were pooled with combinational dual indexes (Supplementary Tables S3 and S5), and 151 bp paired-end reads were obtained using the Illumina HiSeq X.

### 2.4. Bioinformatics pipeline

In the case of analysis for the 12 species, all data from the 151 bp paired-end sequence were trimmed to 150 bp paired-end data using Trimmomatic v.2.0^25^ with the following parameters, ‘CROP : 150’. Because we derived the 17 bases at the 5' end of each raw read from the primer used in the first PCR of MIG-seq, all raw reads were trimmed and filtered using Trimmomatic v.2.0 with the following parameters, ‘HEADCROP : 17 ILLUMINACLIP: TruSeq3-PE-2.fa : 2:30:10 LEADING : 20 TRAILING : 20 SLIDINGWINDOW : 4:15’ (a FASTA format file, TruSeq3-PE-2.fa, contains Illumina adapter sequences, https://github.com/timflutre/trimmomatic/blob/master/adapters/TruSeq3-PE-2.fa). Using BWA mem,^26^ we mapped the trimmed reads of the 12 species to reference genomes of each species (Supplementary Table S6)^11–13^^,^^27–36^ after the sequence alignment/map (SAM) format files were converted to binary alignment map (BAM) and sorted using Samtools version 1.9.^37^ In the case of an analysis to evaluate MIG-seq usefulness for various plant species, we used Samtools depth command to extract coverage depth (DP) information of each locus through genomes from sorted BAM with a set parameter, ‘-d 0’. In this study, nucleotides over 10 DP were defined as the number of sequences, which can be obtained stably by MIG-seq. The variant call is performed using the Samtools mpileup^37^ command with the ‘-d 0’ option. Raw reads of F_2_ population, F_6:7_ RILs, and TWC were filtered and mapped to the reference durum genome^12^ in the same way, as stated above. For the variant calling of the TWC, F_2_ population, and F_6:7_ RILs, we used GATK Haplotype caller v4.1.7.0^38^ to generate a ‘g.vcf’ format file for each sample. We performed joint genotyping using GATK GenomicsDBImport and GenotypeGVCF to create one VCF for each ‘g.vcf’ sample.

### 2.5. Investigating the number of bases that can be stably sequenced, extracting the number of SSRs, and nucleotide diversity in various plant species

For the analysis of the 12 species, the DP value was divided by the amount of raw read data (Gb) in each fastq file and multiplied by 0.5 to obtain the DP value per 0.5 Gb. Krait ^39^ was used to search the number of SSRs in the reference genome for the 12 species and summed the number of SSRs (ACT, TTG, GTG, and GT) appearing in the primers used for the first PCR of MIG-seq. We obtained the genome size from the number of bases in the FASTA file of the reference genomes and the correlation coefficients between the genome size and ‘mapped base count’, which is defined as the number of bases above DP 10. Then, we investigated the correlation between genome size and SSRs. Using VCFtools with the ‘-site-pi’ option, we calculated the nucleotide diversity of each species.

### 2.6. Population structure analysis

After the bioinformatics process, we filtered the vcf file of TWC using VCFtools^40^ with parameters: –max-missing 1 –minDP 5 –minQ 30 –recode –recode-INFO-all. To eliminate SNPs/indels containing heterozygous genotypes completely, the filter option of SnpSift was used with parameter: ‘countHet() = 0’. The filtered VCF was used for Admixture^41^ to estimate individual ancestry. After PLINK 1.90^42^ was used to remove SNPs in linkage disequilibrium with parameter: –make-bed –indep-pairwise 50 5 0.5 –allow-extra-chr, we ran Admixture. The phylogenic tree of TWC was constructed using MEGA7^43^ with SNPs obtained from MIG-seq. For principal component analysis (PCA), the VCF file was further filtered by PLINK 1.90 in linkage disequilibrium with parameter: –make-bed –indep-pairwise 50 10 0.1 –allow-extra-chr, and PCA was performed using PLINK 1.90.

### 2.7. Genetic linkage map construction and QTL analysis

The first VCF file was read by ‘vcfR,’^44^ and genotypes with DP of 10 and DP of 5 or less were converted to missing data for the F_2_ population and the F_6:7_ RIL, respectively. Using handmade R script, individuals with extremely low genotype deficiency (less than 5% and 10% in the F_2_ population and F_6:7_ RILs, respectively) were deleted. For accurate linkage mapping, markers were filtered based on their genotype rate, the percentage of individuals whose genotype was obtained for a marker.

Linkage maps of the F_2_ population and F_6_ RILs were constructed using the R package ‘one map’.^45^ In the F_2_ population, markers were grouped using the ‘group’ function of the ‘Onemap’ package, while in the case of F_6:7_ RILs, markers were separated by chromosome and grouped using the ‘group’ function of ‘Onemap’ package. We calculated linkage distances using Kosambi’s function (Kosambi 1944). The order of markers was determined using the ‘order_seq’ function. QTL analysis was performed by the composite interval mapping (CIM) method using ‘R/qtl’ package.^46^ The threshold of the logarithm of odds (LOD) score was determined by the 1,000 permutation test.

### 2.8. Evaluating the accuracy of heterozygous genotype call

To investigate the amount of raw data and the minimum coverage depth that we can accurately call the heterozygous genotype data in the mapping population, the analysis was performed using the following procedures. First, the Seqkit^47^ sample command was used to extract 0.5, 1.0, 1.5, 2.0, 2.5, and 3.0 million reads from eight replicates of TTW41 and TTW139 data, respectively. These sampled raw data of TTW41 and TTW139, respectively were independently merged to create eight independent replicates of virtual F_1_ data. The amount of data in the virtual F_1_ data is 1, 2, 3, 4, 5, and 6 million reads, respectively. For these data, VCF file was created using the same bioinformatics pipeline as the case of the F_2_ population. Markers used to construct the linkage map of F_2_ were extracted and the missing and error rates for each minimum coverage depth threshold (from 1 to 20) of the markers were calculated using handmade Rscript.

### 2.9. Calculation of nucleotide diversity of all combinations of two accessions in TWC

The number of polymorphisms (SNP/indels) between the two varieties was calculated for all combinations (18,721 combinations) of the TWC raw vcf file filtered by VCFtools^40^ with parameters: –minDP 5 –minQ 30 –recode –recode-INFO-all. The number of SNPs was also calculated for the above vcftools condition with the –remove-indels option. The number of bases that could be sequenced with DP5 or higher was extracted using Samtools depth^37^ command from BAM format files of TWC. Nucleotide diversity was calculated by dividing the number of SNPs between the two accessions by the number of bases with a DP of five or higher.

## 3. Results

### 3.1. Evaluation of MIG-seq performance for various plant species that include crop

**Figure 1:**
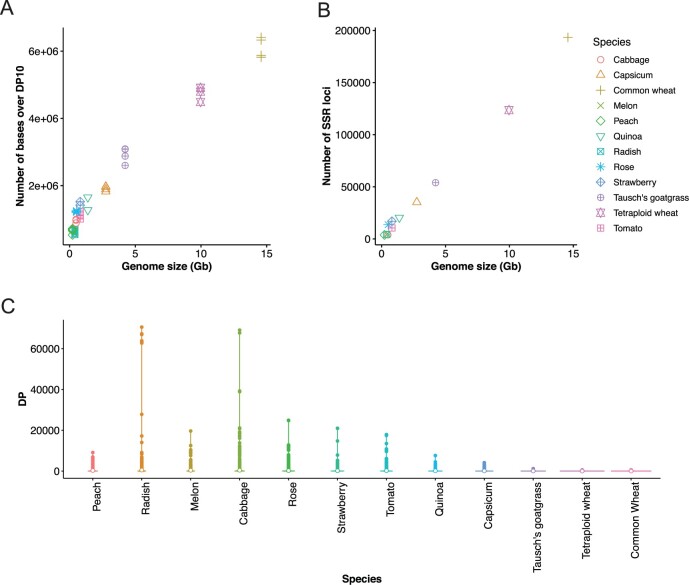
Evaluating the performance of MIG-seq using 12 plant species. (A) Relationship between genome size and the number of mapped base count. (B) Relationship between genome size and number of SSR loci number of SSRs present in the genome that are included in the primers of the first PCR of MIG-seq. (C) Violin plot of coverage depth (DP) of SNP/indel in each species. ‘DP’ is the average coverage depth for each polymorphism between/among accessions.

We could obtain about 264,197,942 of 151 or 150 bp paired-end reads for the 12 species. Raw read counts for each sample ranged from 3,482,874 (about 0.52 Gb) to 10,355,658 (about 1.55 Gb) (Supplementary Table S1). In this study, we investigated the relationship between the ‘mapped base count’, defined as the sum of the number of bases, with DP greater than or equal to 10, obtained from the whole genome, and the genome size calculated from the number of bases in the FASTA file for the 12 species. The results revealed that the mapped base count increased as the genome size increased, and that the common wheat, which has the largest genome size, had the largest mapped base count (Fig. 1A). Mapped base count and genome size were positively correlated (*P*-value < 2.2 × 10^−16^, *R* = 0.986) (Fig. 1A). To verify this result, we used Krait^39^ to investigate the number of SSRs in the FASTA format files of the reference genomes of the 12 species because we expected the number of SSRs to increase with genome size and the number of ISSRs to increase accordingly. Using the results of SSR regions extracted from the reference genome of each species by Krait,^39^ we calculated the total number of SSRs targeted by the primers used in the first PCR of MIG-seq. The number of SSRs was significantly associated with the genome size (*P*-value < 2.1 × 10^−13^, *R* = 0.998, Fig. 1B). The SNPs detected by MIG-seq were distributed across the genome, but the DP values of each SNP/indel significantly varied among species. In wheat, the DP of several SNPs/indels was less than 1,000, but some polymorphisms exceeded 70,000 DP in radish (Fig. 1C, Supplementary Fig. S1). The nucleotide diversity of the 12 plant species ranged from 0.000825 to 0.0111. The rose materials used in this study had high nucleotide diversity, and we detected relatively many SNPs in the rose accessions (Supplementary Table S7).

### 3.2. Admixture analysis, constructing the phylogenetic tree, and PCA using SNP/indels from MIG-seq

We obtained 1,298,184,266 raw reads from two lanes of HiSeq X sequencing. Since the average number of raw reads per sample in TWC was 6,657,355.21, while the number of reads in TTW196 was 1,176,140, only the durum wheat line TTW196 was excluded from subsequent analyses (Supplementary Table S2). After filtering low-quality reads, mapping to the reference genome, and variant calling, we detected 3,848,416 SNP in the unfiltered VCF file. SNPs/indels with heterozygous genotypes and even a single missing data among accessions were removed, resulting in 8,207 polymorphisms (Table 1; 7,794 SNPs and 413 indel polymorphisms). We used 8,207 SNP/indels for PCA, while 7,794 SNPs were used for constructing phylogenic trees by MEGA7. For Admixture analysis, 8,207 SNP/indels were filtered using PLINK 1.90, resulting in 4,947 SNP/indels (Supplementary Table S8).

**Table 1:** Number of SNPs and indels of each chromosome in the F_2_ population, F_6:7_ RILs, and TWC

Chromosome	SNPs and indels in the F_2_ population	SNPs and indels in the F_6:7_ RILs	SNPs and indels in the TWC
1A	237	224	657
1B	238	227	427
2A	369	276	589
2B	334	313	524
3A	188	152	641
3B	262	304	667
4A	169	170	773
4B	157	246	577
5A	185	156	688
5B	168	223	510
6A	256	230	639
6B	241	234	403
7A	298	232	620
7B	155	197	532
All chromosome	3257	3148	8207

**Figure 2:**
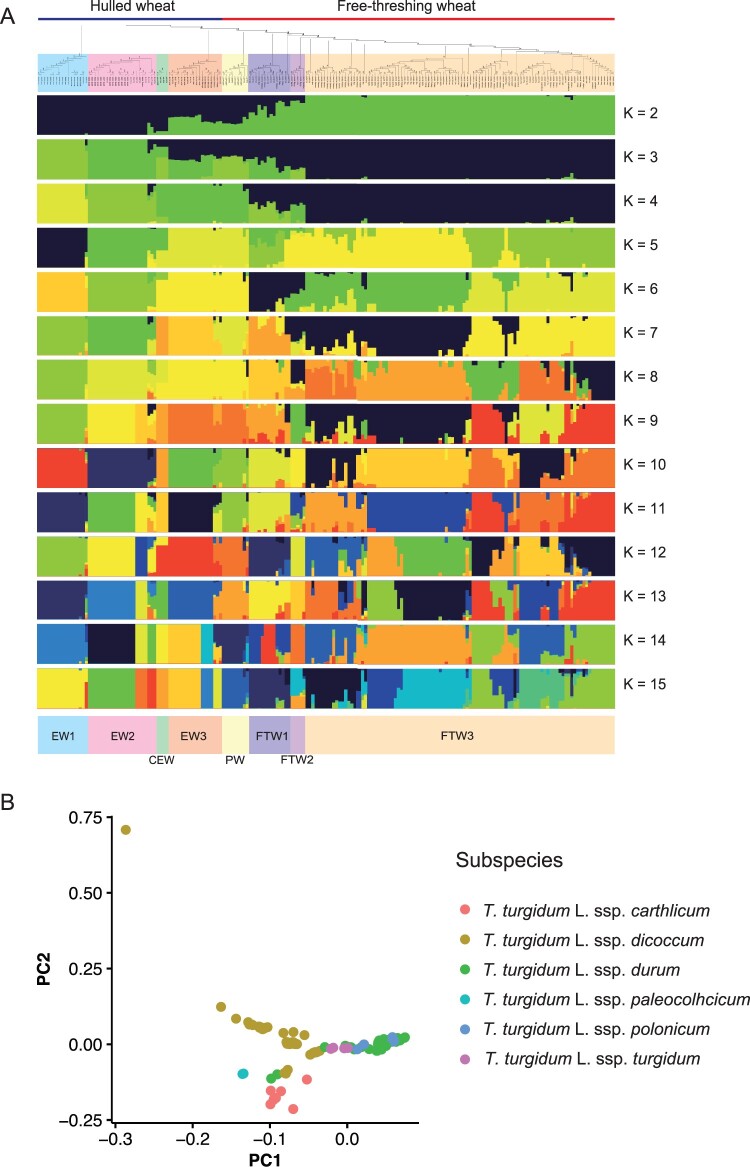
Admixture analysis, phylogenetic tree, and principal component analysis of tetraploid wheat population using SNP/indel markers obtained by MIG-seq. (A) Phylogenic tree and admixture analyses in TWC. EW1, EW2, and EW3 consisted mainly of emmer wheat from the Soviet Union and Iran, Spain, Ethiopia, and India; CEW included all Colchis emmer wheat; EW2 included all emmer wheat from Egypt. PW includes Persian wheat. FTW1 consists mainly of rivet wheat, durum wheat, and Polish wheat. FTW2 contains durum wheat from Egypt. FTW3 contains durum wheat from various countries. (B) PCA of TWC using SNP derived from MIG-seq.

Based on the results of the phylogenetic tree, hulled wheat was divided into four groups (EW1, EW2, CEW, and EW3) and free-threshing wheat was divided into four groups (PW, FTW1, FTW2, and FTW3) (Fig. 2A, Supplementary Figs S2 and S3). EW1, EW2, and EW3 were mainly composed of emmer wheat from USSR/Iran, Spain, and Ethiopia/India. CEW included all Colchis emmer wheat. For free-threshing wheat, PW includes Persian wheat. FTW1 was mainly composed of rivet wheat, durum wheat, and Polish wheat. FTW2 contained durum wheat derived from Egypt. Wheat accessions of FTW3 were durum wheat from various countries, and there seem to be several clades, but the bootstrap percentages for the first 10 branches of FTW3 were less than 30, so these were classified into one clade. Subspecies did not separate the PCA results, and some plots between subspecies were quite close and/or overlapped, but the same species tended to be located relatively close to each other (Fig. 2B). It was found that one sample of emmer wheat with large PC2 was TTW7 (KU-117), which is an accession that belongs to EW1 in the phylogenetic tree. This accession belongs to a different branch from the other 16 EW1 accessions (Supplementary Figs S2 and S3). As for admixture results, cross-validation revealed that the optimum k was 13 (Supplementary Fig. S4). The admixture results were broadly consistent with the phylogenic tree results, although finer clades existed within EW2 and FTW3. In phylogenetic trees, durum wheat clades include rivet and Polish wheat, consistent with the previous study.^12^ This finding supports the reliability of the polymorphism information obtained by MIG-seq.

### 3.3. Relationship between DNA concentration and amount of acquired data

**Figure 3:**
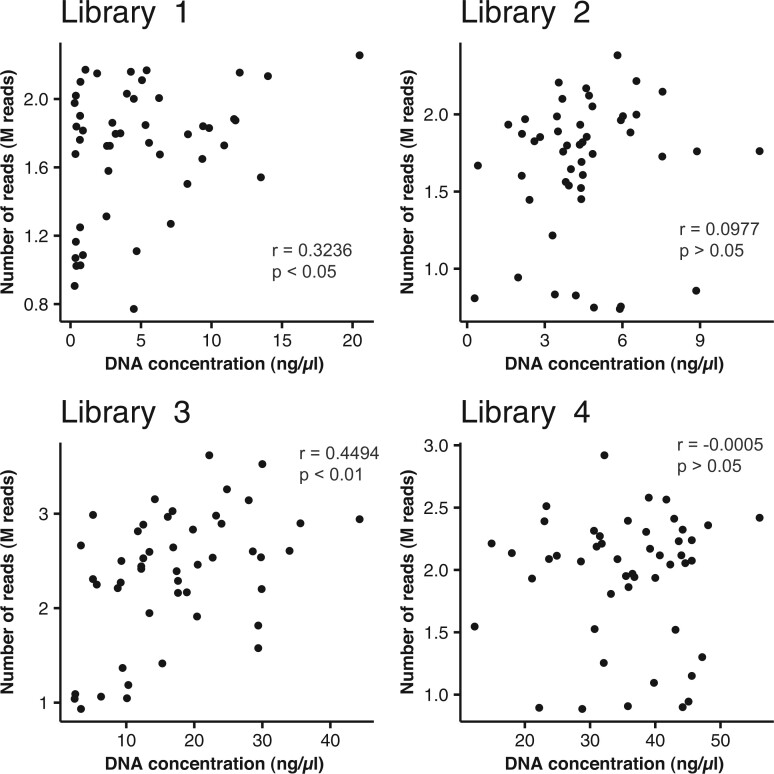
Scatter plot of DNA concentration *vs*. the number of reads obtained in F_6:7_ RILs. Library 1, Library 2, Library 3, and Library 4 indicate four independent MIG-seq libraries of F_6:7_ RILs consisting of 48 samples.

Various DNA concentrations (from 0.291 ng/μl–56.0 ng/μl; Supplementary Table S3) of each line of F_6:7_ RILs were used for 1st PCR of MIG-seq. Then, we examined the relationship between the amount of DNA input and the number of raw reads obtained for each line used for linkage mapping. A weak positive correlation was observed between DNA concentration and number of raw reads for Library 1 and Library 3, but no significant positive correlation was observed for Library 2 and Library 4, suggesting that DNA concentration does not strongly affect the number of raw reads (Fig. 3). There was no positive correlation between the percentage of genotypes obtained in each individual of the F_6_ generation and DNA concentration used for the first PCR of MIG-seq (Supplementary Fig. S5).

### 3.4. Linkage map construction of F_2_ population and F_6:7_ RILs

In the parental lines of F_2_ and F_6:7_ RILs, 43,110,852, 41,753,354, 8,298,912, and 15,331,396 raw reads were obtained for TTW41, TTW139, TN26, and TN28, respectively. The number of SNPs/indels between the parental lines that exhibited the same genotype and no missing values in eight replicates of the parental lines was 4,759 and 2,468 between the parental lines of the F_2_ population and the F_6:7_ RIL, respectively. The SNPs/indels that indicated the same genotype within the non-missing data and had polymorphisms between parental lines were 17,040 and 20,774 in F_2_ and F_6:7_, respectively. In the case of merging data from eight iterations of the parent line, we detected 46,737 and 29,031 SNPs/indels between the parental lines of the F_2_ population (TTW41 and TTW139) and the parental lines of F_6:7_ RILs (TN26 and TN28), respectively.

**Figure 4:**
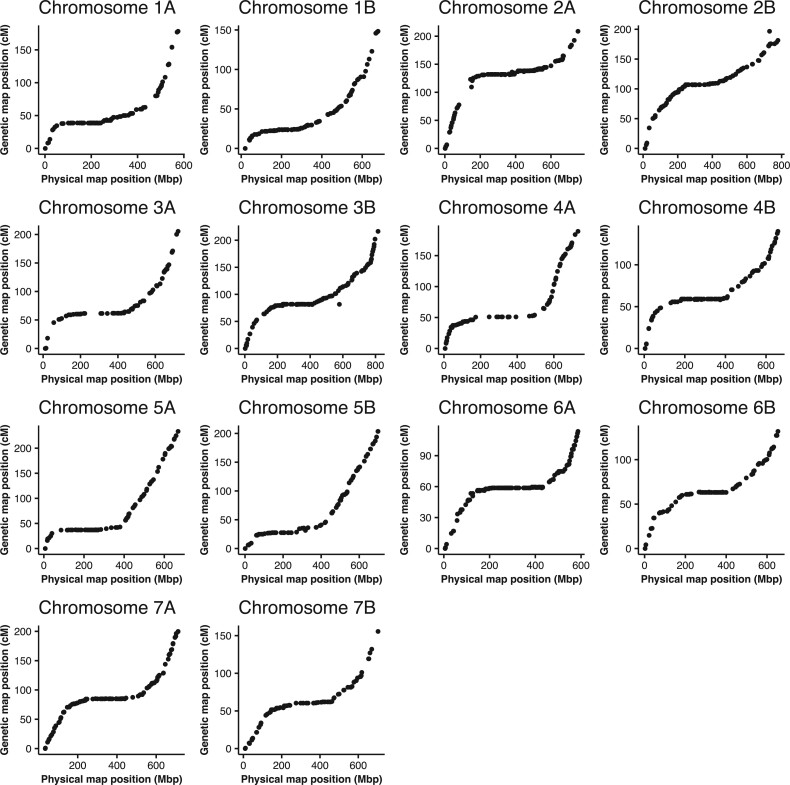
Dot plot of physical and genetic distances of markers on each chromosome for F_6:7_ RILs. The x-axis indicates physical map position of markers and the y-axis indicates genetic map positions of markers.

We obtained 344,498,060 and 498,607,150 raw reads in F_2_ population and F_6:7_ RILs, respectively. Individuals with a high rate of missing genotype data in the segregating population were removed from subsequent analyses (data of one individual of the F_2_ population and one line of F_6:7_ RILs were removed). Considering the genotyping rate for markers (Supplementary Fig. S6), we extracted markers for which more than 97% of the individuals in the F_2_ population and more than 95% of the lines in the F_6:7_ population were genotyped. From these markers, 3,601 and 3,175 markers could be genotyped in the F_2_ population and F_6:7_ RILs, respectively. For F_6:7_ RILs, heterozygous genotype data were converted to missing data. After grouping by OneMap ‘group’ function, we removed non-linked markers. We successfully generated a linkage map consisting of 3,257 and 3,148 markers in the F_2_ population and F_6:7_ RILs, respectively (Fig. 4, Table 1, Supplementary Figs S7–S9, Supplementary Data S1 and S2). The total length of the linkage map was 5,022.0 cM and 2,521.6 cM, and the average distance of loci was 1.54 cM and 0.80 cM in the F_2_ population and F_6:7_ RILs, respectively (Supplementary Tables S9 and S10). Comparing the genetic position and the physical position of the durum reference genome in both cases of linkage map in F_2_ population and F_6:7_ RILs, suppression of recombination near the centromere was observed, and the frequency of recombination increased near telomeres, as expected (Fig. 4). There were a few regions with few markers, such as 2A and 3A, but we obtained a map with marker information across the entire genome (Supplementary Fig. S10). For the F_6:7_ RILs, distortion of the segregation ratio was observed in the short arm of chromosome 5B (Supplementary Data S2).

### 3.5. QTL analysis for heading date

**Figure 5:**
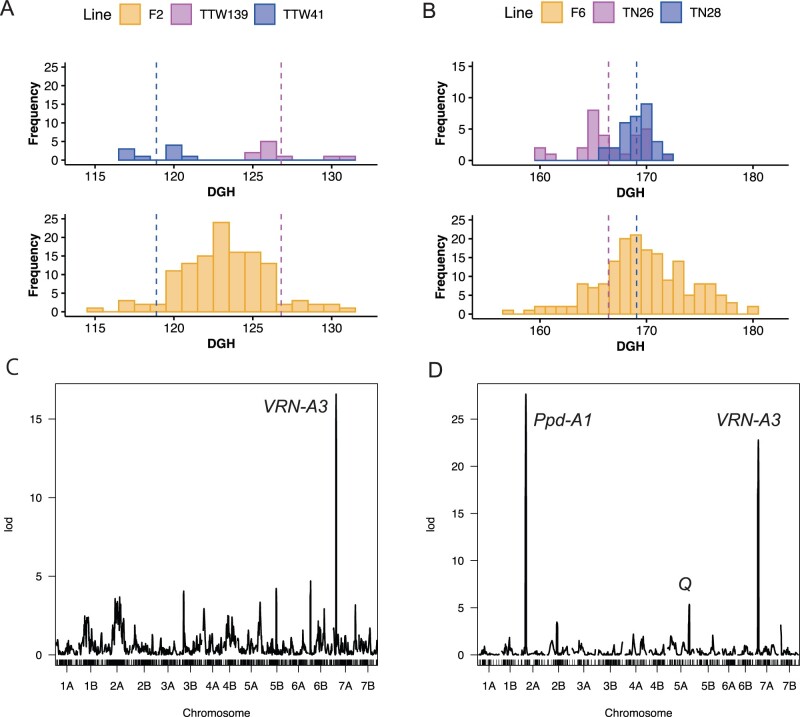
Phenotype data of F_2_ population and F_6:7_ RILs and their parents, and LOD scores of QTLs of F_2_ population and F_6:7_ RILs. (A) A histogram of DGH in the F_2_ population and its parental lines. (B) A histogram of DGH in the F_6:7_ RILs and its parental lines. Dotted lines mean the average DGH of two parental lines. (C) LOD scores of QTLs in the F_2_ population. (D) LOD scores of QTLs in F_6:7_ RILs.

Figure 5 depicts the results of the QTL analysis. The DGH for the F_2_ population and F_6:7_ RILs ranged from 115 to 131 and from 157 to 180.5, respectively (Fig. 5A and B). The average DGH of TTW41, TTW139, TN26, and TN28 were 118.89, 126.80, 166.43, and 169.07, respectively. After 1,000 permutation tests, the LOD score thresholds were 6.09 and 5.09 for the F_2_ population and F_6:7_ RILs, respectively. In the F_6:7_ RILs, QTLs exceeding this threshold were detected on chromosomes 2A, 5A, and 7A, with LOD scores of 27.65, 5.35, and 22.78, respectively (Fig. 5C and D, Table 2). In the case of chromosome 2A QTL, the *Ppd-A1* gene^48^ was located between the two markers (positioned between two markers: 2A_36,075,870 and 2A_41,495,996) flanking the peak of the LOD score. Similarly, the QTL of chromosome 7A and the QTL of chromosome 5A showed that the *VRN-A3*^21^^,^^49^ (positioned between two markers: 7A_68,283,463 and 7A_76,232,738) and *Q* genes^50^ (positioned between two markers: 5A_604,404,784 and 5A_622,238,335), which affect the time of heading and flowering, were located between the two markers that flank the peaks of LOD score. (Supplementary Fig. S11). The similarity of the results of QTL analysis to SSR marker analysis in our previous study^49^ indicates the accuracy of genotype data obtained by MIG-seq in wheat. In this study, a QTL at 7A chromosome was detected in this F_2_ population, as expected, since we assessed the effect of the *VRN-A3* gene on heading date using the same F_2_ population derived from a cross between TTW41 and TTW139 in our previous study.^20^ The peak position of this QTL was not between the adjacent markers of *VRN-A3*.

**Table 2:** QTLs for heading date in F_2_ population and F_6:7_ RILs using linkage map by MIG-seq

Population	Chromosome	Closest marker	Position (cM)	Peak position (cM)	LOD	Additive effect	*R* ^2^ (%)
F_2_	7A	7A_62760233	40.00–49.00	44.00	16.57	2.33	41.1
F_6:7_	2A	2A_36075870	36.00–45.00	41.00	27.65	−2.53	34.8
F_6:7_	5A	5A_604404784	190.00–194.00	192.00	5.35	0.96	7.0
F_6:7_	7A	7A_68283463	19.00–28.51	25.00	22.78	2.38	30.1

Plus values of additive effect mean the early flowering effect of TN26 allele in F_6:7_ RILs, while that in F_2_ population means the early flowering effect of TTW41 allele.

### 3.6. Validating the amount of data for accurate heterozygous genotype calls

**Figure 6:**
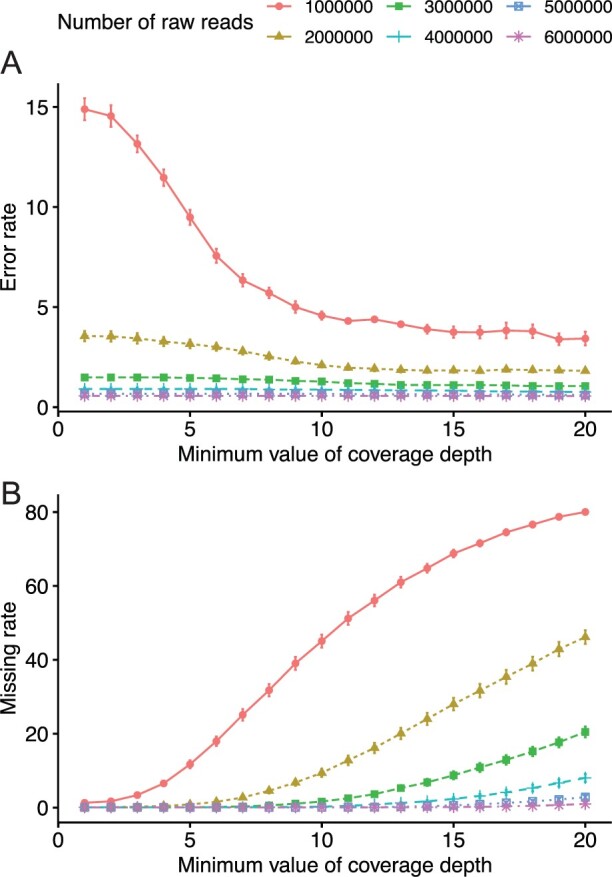
Assessing accuracy in calling heterozygous genotypes using data from virtual F_1_ between TTW41 and TTW139. (A) Genotyping error rate per minimum coverage depth and number of raw reads. The X-axis shows the minimum coverage depth required for genotype calling, and the Y-axis error rate shows the percentage of genotypes called as homozygous when they are called. The color line is changing depending on the amount of raw data. (B) Missing rate of genotypes per minimum coverage depth and number of raw reads.

This study examined the amount of data and minimum DP threshold at which heterozygosity could be accurately estimated for the markers used in F_2_ linkage map construction. For 1 million reads, the error rate (the rate at which heterozygous loci are incorrectly identified as homozygous) was higher than for more than 2 million reads, and even with a minimum DP of 10, 4.58% of the data was in error (Fig. 6A). Increasing the minimum DP beyond 10 did not significantly improve the error rate. As the amount of data increased, the percentage of missing data reduced; for a minimum DP of 10, the percentage of missing data was less than 1% (0.24% missing) for more than 4 million reads (Fig. 6B). If about 4 million reads with a minimum DP of 10 or more was obtained, the heterozygous genotype with high accuracy could be expected. Alternatively, even with 6 million reads, the error rate did not reach zero, and no significant improvement of the error rate for 5 million reads was observed.

### 3.7. Distribution of nucleotide diversity between two accessions in TWC

**Figure 7:**
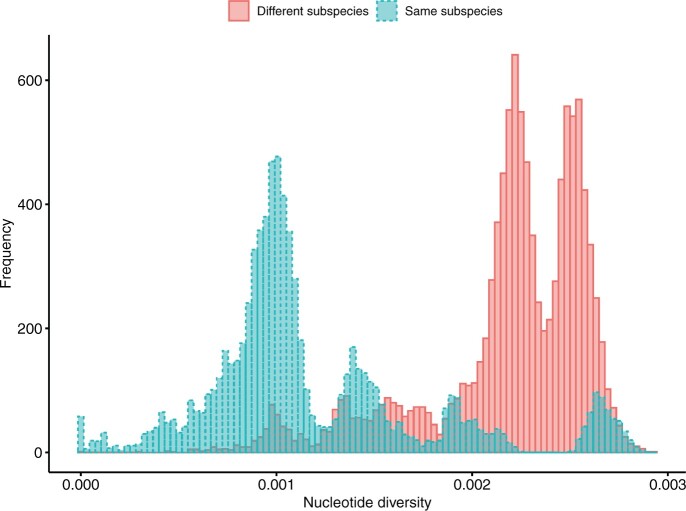
Bars surrounded by dashed line and solid line indicate nucleotide diversity between two accessions of same subspecies and that between two accessions of different subspecies, respectively.

To clarify the range of material wheat combinations for which a linkage map of > 1000 markers by MIG-seq is expected to be constructed, the distribution of the number of polymorphisms when using a collection of tetraploid wheat varieties was investigated. As the number of polymorphisms between two accessions is considered to be determined by the nucleotide diversity and number of common loci between the two accessions (number of bases with DP greater than 5 for both accessions), nucleotide diversity and common loci between the two accessions were calculated for all combinations selected from 194 varieties, two accessions at a time (18,721 combinations) (Fig. 7, Supplementary Figs S12 and S13 and Data S3). We found that the number of polymorphisms was strongly correlated with nucleotide diversity in the present data set, while there was no positive correlation between the number of polymorphisms and the common loci between the two accessions (Supplementary Figs S13 and S14). On the other hand, a weak negative correlation was observed between nucleotide diversity and common loci between the two accessions (Supplementary Figs S13 and S14). As a result, we expect that the relationship between nucleotide diversity and the number of SNPs is not perfectly linear in the present data set. However, when data is extracted based on number of raw reads (mean number of raw reads between two accessions), the number of polymorphisms was almost linearly correlated to the nucleotide diversity (Supplementary Fig. S15). Therefore, by using a strong proportional relationship between nucleotide diversity and the number of SNPs in TWC, the number of markers that can be used to construct a linkage map between any two accessions as parents can be approximated from the number of markers of the linkage map constructed in this study and nucleotide diversity between the parental accessions, only for the data of TWC. The nucleotide diversity between TTW41 and TTW139 and that between TN26 and TN28 was 0.002548 and 0.002758, respectively. Then, assuming simple linearity between number of polymorphisms and nucleotide diversity, it was predicted that a linkage map with over 1,000 markers could be created for accession combinations with a nucleotide diversity of at least 0.000782, which is the value of 1,000 divided by 3,257 (number of markers in linkage map of F_2_ population) multiplied by 0.002548 (the nucleotide diversity between TTW41 and TTW139), although it should be noted that not all polymorphisms between the parental accessions can be used as markers of low missing rate. The rate of combinations above this value (0.000782) was 82.76% between accessions in the same subspecies and 99.58% between accessions in different subspecies.

## 4. Discussion

In this study, we applied MIG-seq to crops to detect polymorphisms for genetic analyses for the first time to the extent of our knowledge. From the results of evaluating the effectiveness of MIG-seq concerning genome size, we discovered that the number of bases that can be sequenced is associated with genome size. Additionally, the number of SSRs targeted by the first PCR of MIG-seq in the genome of species used in this study indicated a significant correlation with the genome size. Therefore, we have clarified that a relatively large number of loci could be sequenced by MIG-seq, and sufficient SNPs/indels could be obtained for genetic analysis in species with large genome sizes, such as wheat. Alternatively, we could not sequence more loci for plant species with a small genome size of less than a few Gb than species with a large genome. Although few polymorphisms may limit the accuracy of genetic analysis, the number of polymorphisms depends not only on the number of loci that can be sequenced but also on the genetic distance between accessions. Numerous polymorphisms could be obtained even in plant species with small genome sizes by MIG-seq using a sample with high nucleotide diversity (π > 0.01), such as the rose used in this study.

When we examined the DP of each polymorphism obtained, we discovered a significant variation in the DP of each SNP/indels in species other than wheat. In some cases, we found regions with a DP of over 60,000 in radish and cabbage, which are too much DP for detecting polymorphisms (Fig. 1C, Supplementary Table S7). This variation may be attributed to the difference in amplification efficiency at each locus or amplification of repetitive sequences, such as retrotransposons, in the first PCR of a MIG-seq. Since over-sequencing the same locus more than 1,000 times is unlikely to improve the accuracy of genotype calling, the smaller the variation in the depth of each SNP, the more efficiently the genotype can be determined. Therefore, suppressing this variation by MIG-seq can result in more efficient detection of polymorphisms at a relatively low cost in any species other than wheat.

From the results of MIG-seq for F_6:7_ RILs, we showed that quantification of sample DNA is unnecessary to construct an NGS library by MIG-seq. In GBS and ddRAD-seq methods, the first step of library construction is restriction enzyme-based, which requires a certain amount of high-quality DNA, and thus either extraction method of high-quality DNA or the cost of DNA purification is required. In MIG-seq, the first reaction is a PCR, so the result is less affected by the quality of DNA. In this study, we could not evaluate the quality of DNA, but we succeeded in constructing a library using DNA obtained using a simple DNA extraction method without using phenol proposed by Zheng et al.^23^ in the F_2_ population of tetraploid wheat. Also, in MIG-seq, we discovered that sequence data could be obtained if a certain amount of DNA could be input during the first PCR. Furthermore, we observed the amount of data did not increase proportionally with the DNA concentration. Therefore, we assumed that it is unnecessary to precisely normalize the DNA concentration using DNA-specific fluorescent dyes, although the lowest DNA concentration in this study was 0.291 ng per 7 μl, at which the first PCR could be amplified, and it may need to be confirmed in the future whether the first PCR can be performed at lower concentrations. These results indicate that the main advantage of MIG-seq is that it eliminates the need for measurement and normalization of DNA concentration for each sample, thus saving cost and time for those experimental operations.

Our results indicated that the number of polymorphisms obtained by MIG-seq in wheat is equal to or slightly less than that of GBS methods reported in previous studies^15^^,^^51^^,^^52^. Although the wheat materials are different in each study, they reported that the numbers of GBS markers that could be genotyped in 80, 91.4, and 95% or more of the individuals were 8,505, 4,662, and 2,975, respectively. In contrast, in this study, we obtained 6,482 and 4,548 markers at 80 and 90% lines in F_6:7_ RILs, and 6,609 and 4,866 markers at 80 and 90% individuals, respectively, in the F_2_ population (Supplementary Fig. S6). The crosses used for constructing the linkage map in this study were combinations of two lines with relatively high nucleotide diversity among TWC, and therefore, depending on the crossing combination, polymorphisms of 3,000 or more may not be obtained. Although the results of the calculation of the nucleotide diversity between the two cultivars indicated that the linkage map could be constructed with over 1,000 markers in most of the tetraploid wheat combinations (more than 82.76% for combinations between the same subspecies) it is necessary to verify the applicability of this method by studying the nucleotide diversity before constructing the linkage map. In the case of population structure analyses of TWC, we detected 8,207 SNP/indels with no missing genotypes. The number of polymorphisms detected in TWC is enough for population structure analysis. Considering the simplicity of DNA adjustment, we suppose that MIG-seq is a high-throughput method, which can be easily applied to larger populations, for example, to easily select core collections from populations of more than 1,000 individuals.

The genotype data from MIG-seq, like other NGS-based methods, may contain some error data. In the linkage map of Tausch’s goatgrass generated by GRAS-Di, the number of markers in the linkage map was significantly high but the total length of the genetic map was extremely longer than that constructed from non-GBS based markers^53^; over 800 cM for each chromosome.^9^ It is reasonable to assume that this is not 800 cM, but error data. In this study, the linkage map was longer in the F_2_ population than in the F_6:7_ RILs, although the effect of having different parents in the F_2_ population and the F_6:7_ RILs had to be considered. This is because the F_2_ population has more heterozygous loci than the F_6:7_ RILs (Supplementary Fig. S8 and Data S1 and S2). If only reads from one parent chromosome were sequenced by chance, the locus would become homozygous, causing false double recombination. Double recombination, in which a heterozygote is inserted into a chromosomal region with a homozygous genotype was also observed (Supplementary Fig. S8 and Data S1 and S2). This could be due to sequencing errors, such as the false detection of fluorescence or index hopping in the sequencing step. In fact, in the hypothetical F_1_ data generated using the F_2_ parental line data, the error rate (error in which a genotype that should be heterozygous is determined to be homozygous) did not become zero even when the number of reads was increased to 6 million. Therefore, it is necessary to consider that a certain amount of error is always included in the analysis. In the case of QTL analysis, such as this study, a small amount of error data will not have a significant impact on the results of the analysis. However, more accurate genotype information may be required to determine the order of scaffolds in the *de novo* assembly of the genome. Since the cost of creating a library for MIG-seq is low, it is possible to increase the number of reads to be acquired and call genotypes or to sequence each strain multiple times and use only the data that match multiple times.

In this study, a clear distortion of the segregation ratio on chromosome 5B was also observed in the linkage map of F_6:7_ RILs, which was also observed in our previous study (Nishimura et al.^49^), where the F_5_ population derived from cross between TN26 and TN28 was genotyped using SSR markers (Supplementary Data S4). Therefore, it is not considered a bias caused by the MIG-seq method.

In this study, we discovered that genome size is associated with the number of bases that can be sequenced by MIG-seq, and as a result, a relatively large number of SNPs can be detected in wheat. Genotyping data with over 3,000 markers and a low defective rate could be obtained without precise normalization of DNA concentration between emmer wheat and durum wheat, and the possibility of constructing a linkage map of more than 1,000 markers with many tetraploid wheat combinations was shown. These results show that MIG-seq can be used for high-throughput genotyping of wheat.

## Supplementary Material

dsac011_Supplementary_Data
