# Supplementary material for: MIG-seq is an effective method for high-throughput genotyping in wheat (Triticum spp.)
Source: DNA Res. 2022 Apr 12;29(2):dsac011. doi: 10.1093/dnares/dsac011 (PMC9035812; doi:10.1093/dnares/dsac011)
Supplement: dsac011_Supplementary_Data [file dsac011_supplementary_data.zip › Supplementary Figures.pdf]

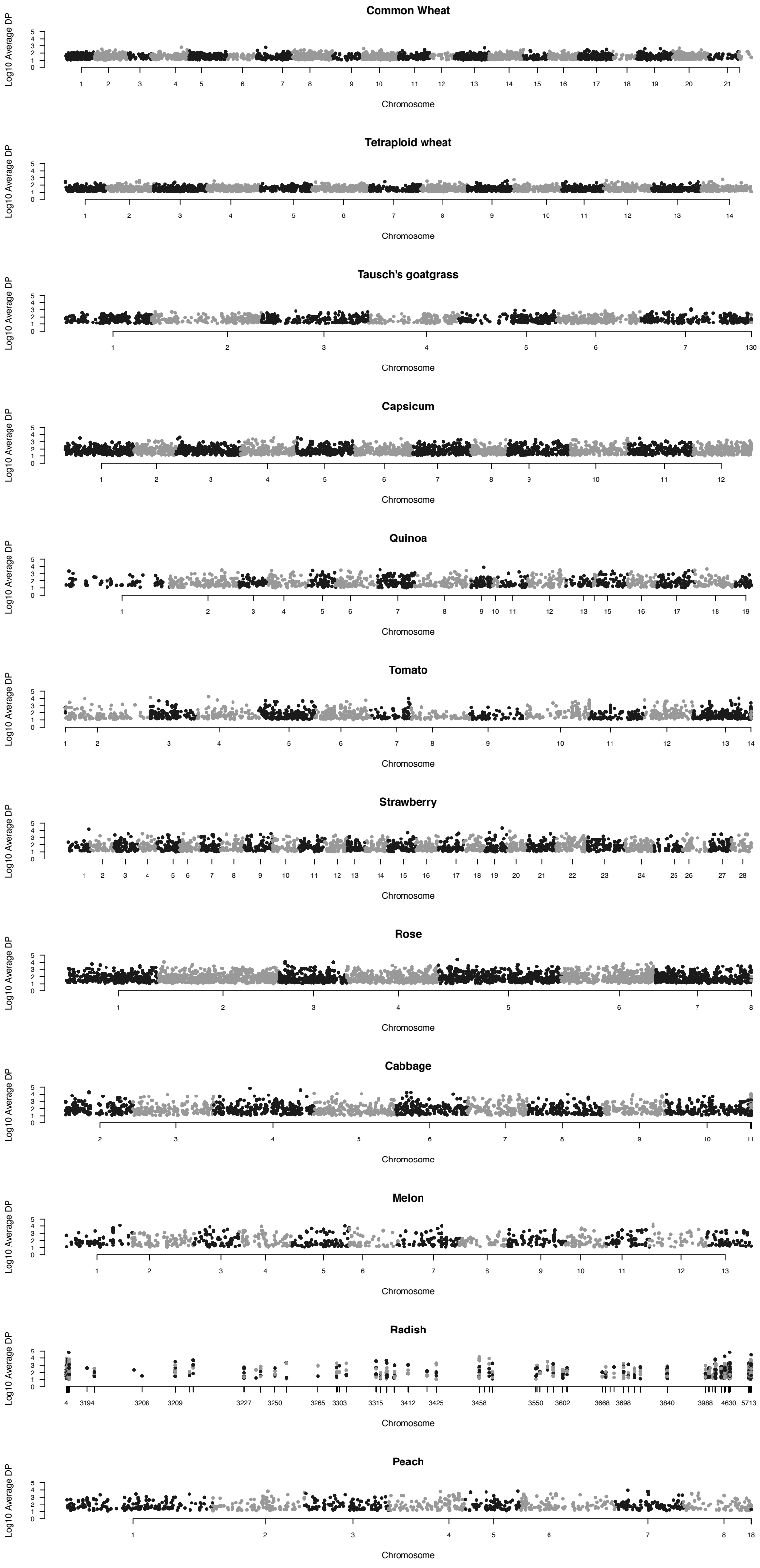

Supplementary Figure S1. Distribution of SNP/indel DP obtained by MIG-seq in various plant species.
The x-axis represents the chromosome and position of SNP/indels and the y-axis represents the LOG10 of the average DP between/among accessions at each SNP/indel.
.

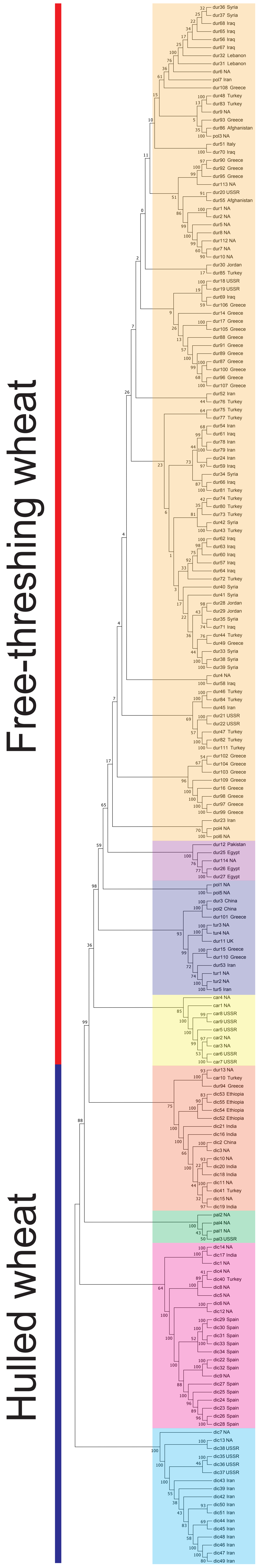

Supplementary Figure S2. Phylogenetic trees that do not reflect genetic distance in branch lengths by the neighbor-joining method using SNPs obtained by MIG-seq.

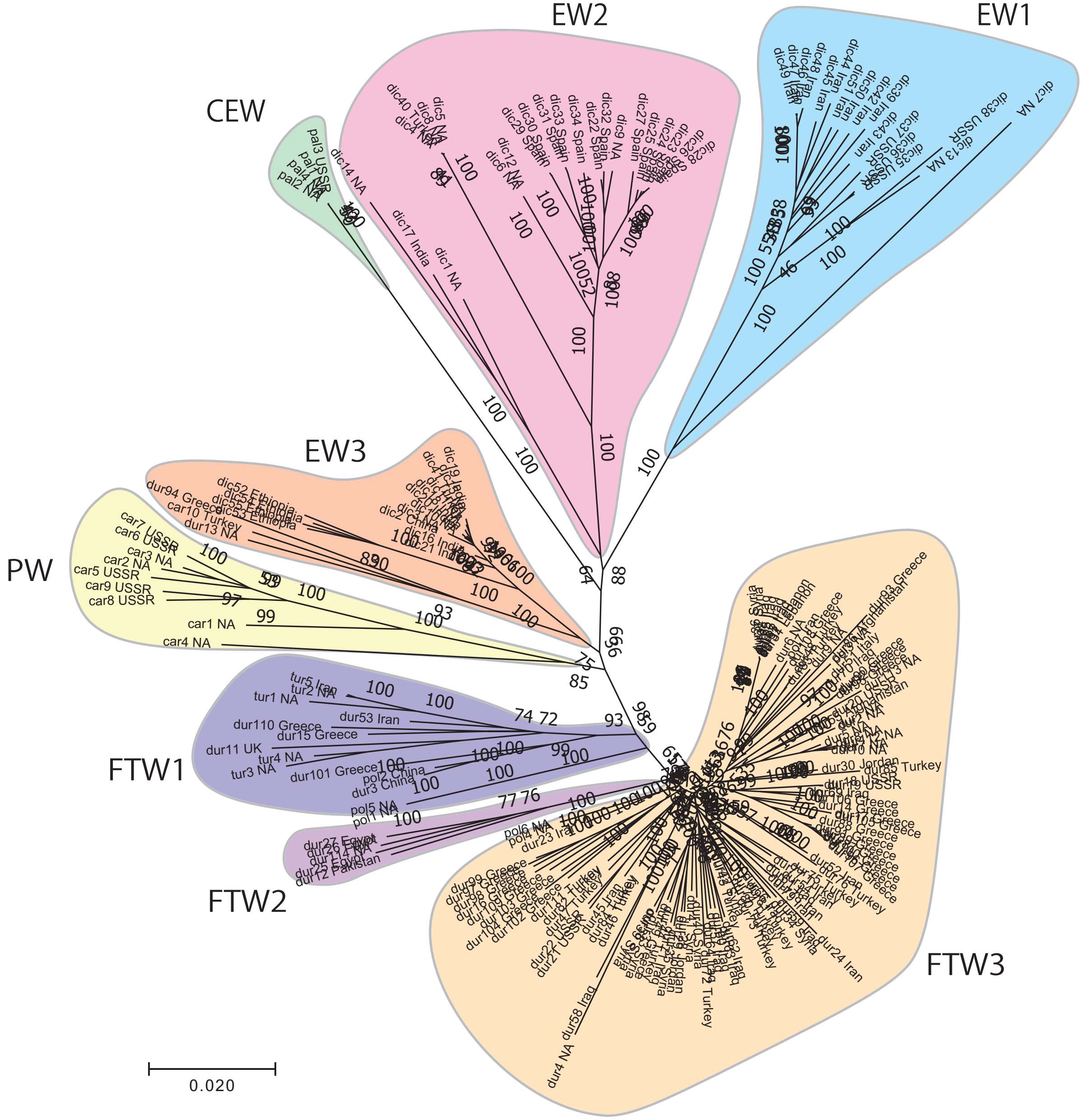

Supplementary Figure S3. Phylogenetic tree reflecting genetic distance in branch lengths by the neighbor-joining method using SNPs obtained by MIG-seq.

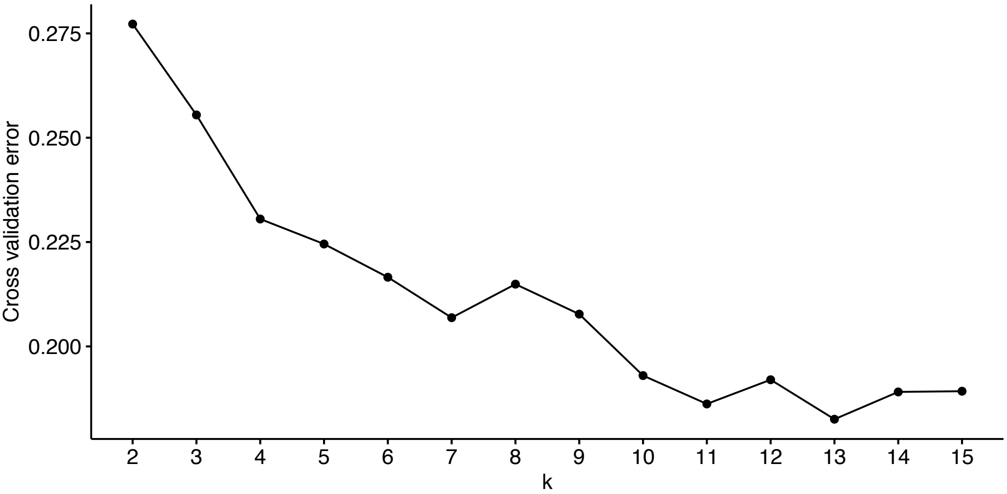

Supplementary Figure S4. The optimal k value by cross-validation
X-axis and y-axis indicate k value and cross-validation error, respectively. The optimal k is 13, where the cross-validation error is minimum.

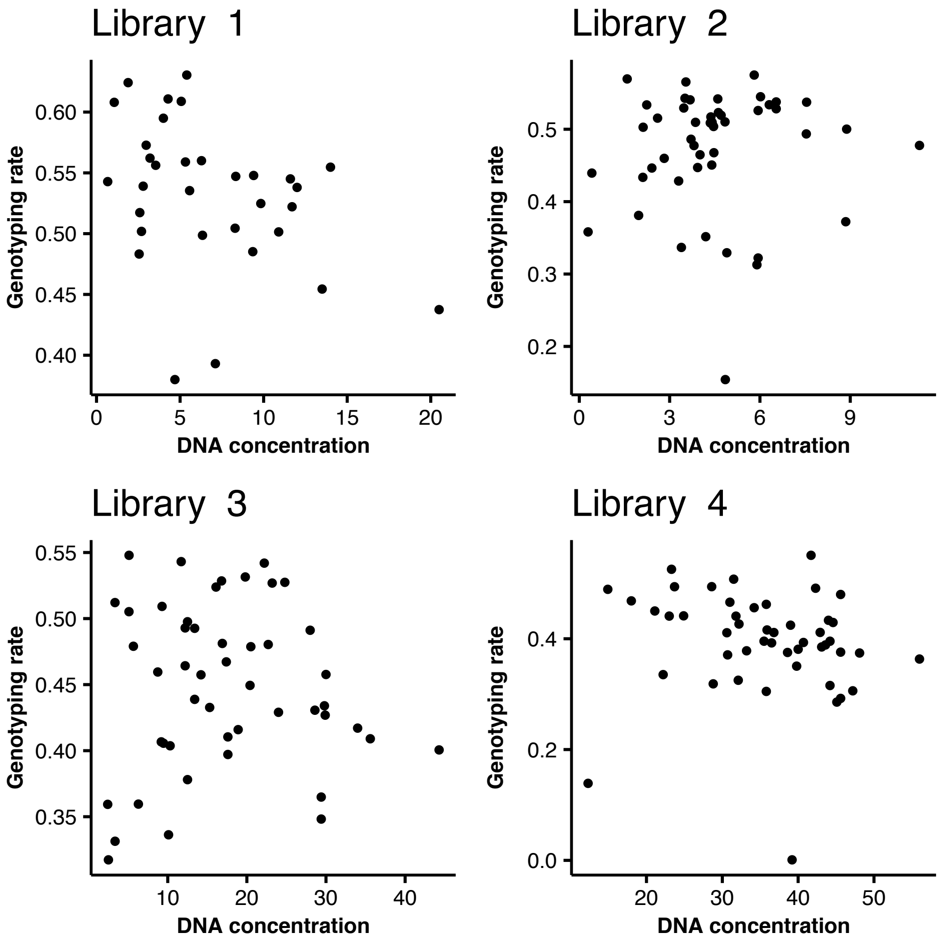

Supplementary Figure S5. Scatter plot of DNA concentration *vs*. genotyping rate obtained in $F_{6:7}$ RILs. Library 1, Library 2, Library 3, and Library 4 indicate four independent MIG-seq libraries of $F_{6:7}$ RILs consisting of 48 samples.

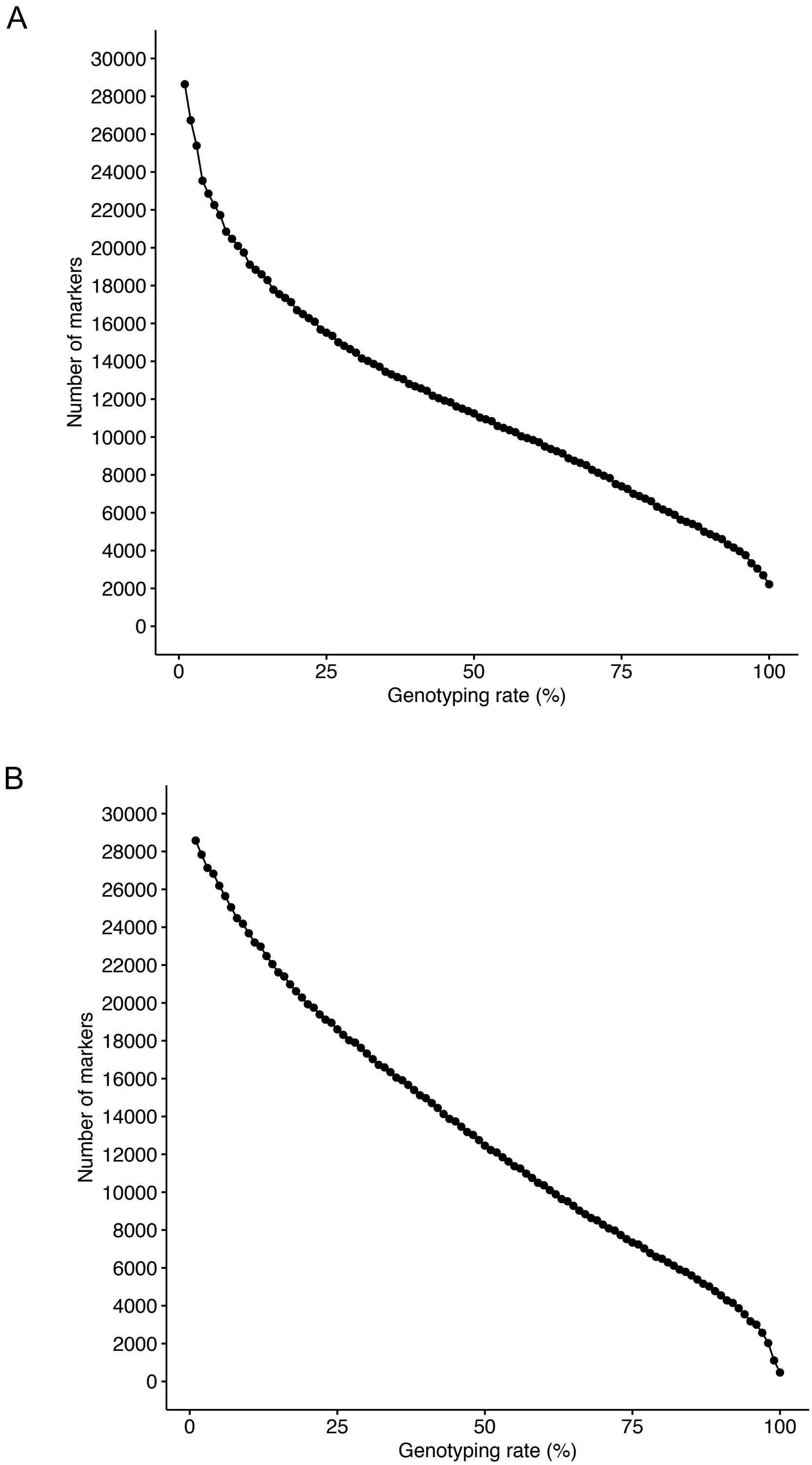

Supplementary Figure S6. Relationships of genotype rate of each polymorphism and number of markers in F_2 and $F_{6:7}$ population
a) F_2 population b) $F_{6:7}$ population

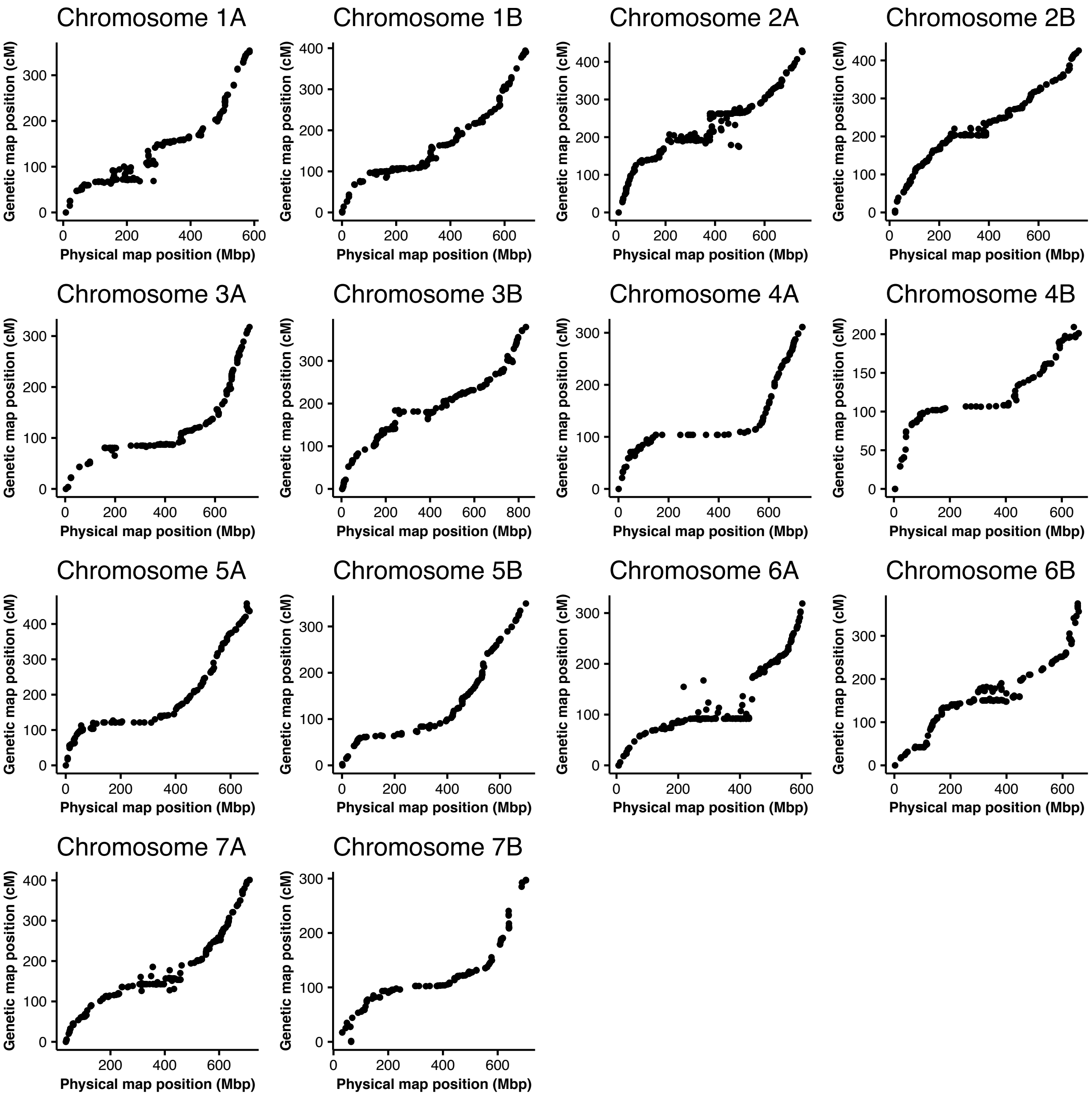

Supplementary Figure S7. Dot plot of physical and genetic distances of markers on each chromosome for F_2 population derived from a cross between TTW41 and TTW139.

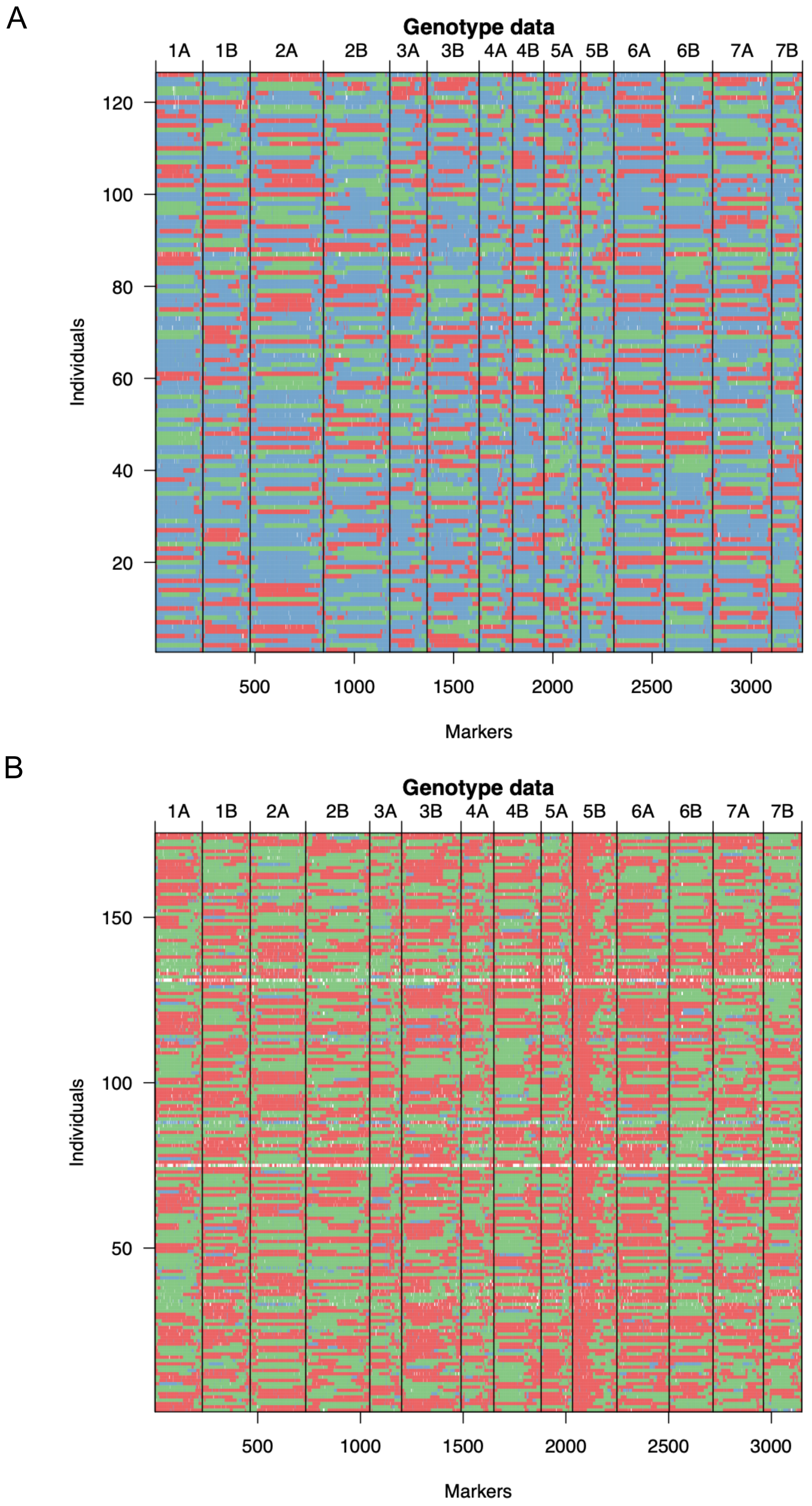

Supplementary Figure S8. Graphical genotypes of F_2 population and $F_{6:7}$ RILs.
a) F_2 population. Green, red, and blue cell indicate homozygous of TTW41, homozygous of TTW139, and heterozygous genotype.
b) $F_{6:7}$ population. Green, red and blue cell indicate homozygous of TN26, homozygous of TN28, and heterozygous genotype.

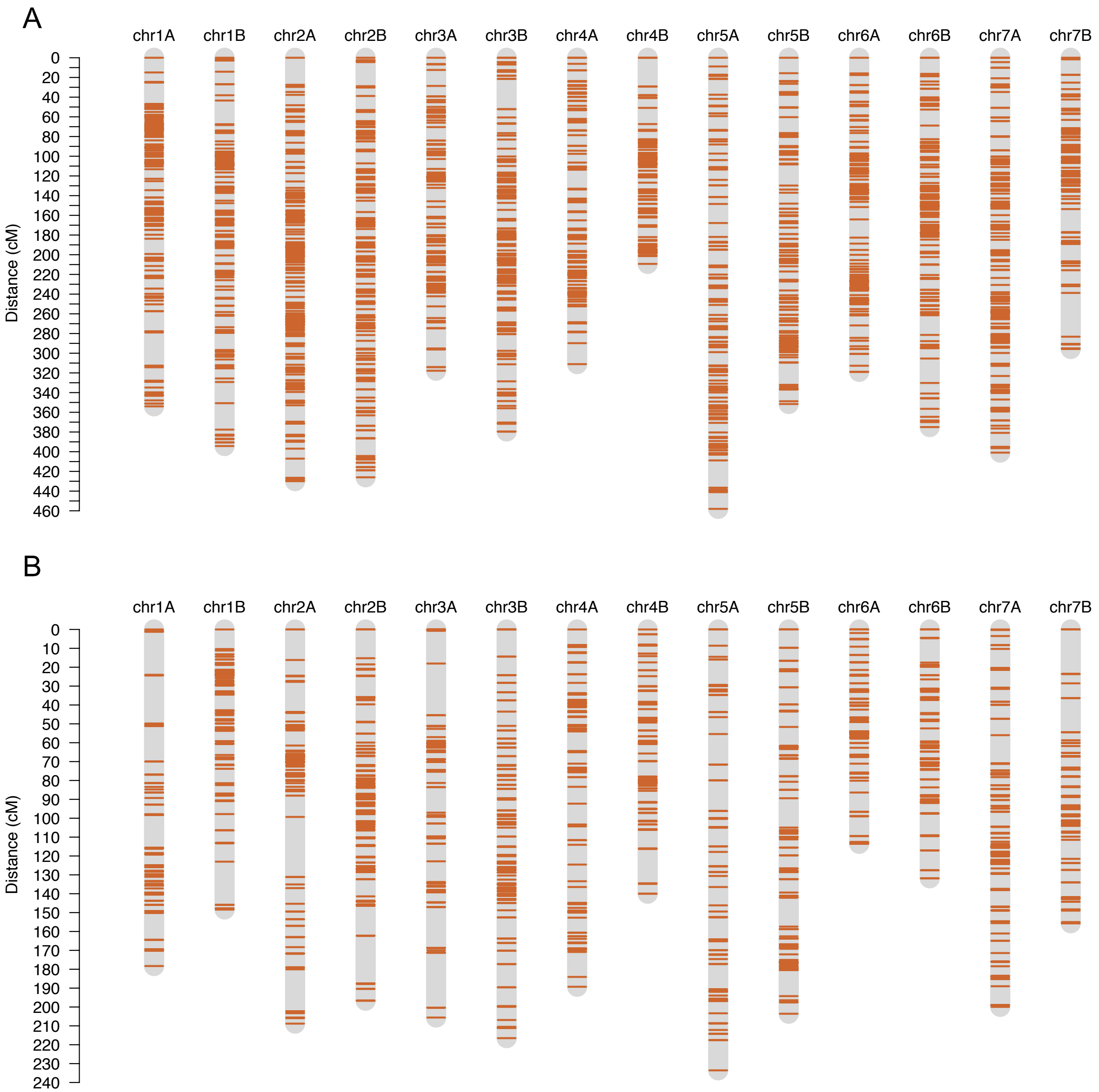

Supplementary Figure S9. Linkage map of F_2 population and F_6 RILs constructed by MIG-seq. a) Linkage map of an F_2 population derived from a cross between TTW41 and TTW139, b) Linkage map of $F_{6:7}$ RILs derived from a cross between TN26 and TN28. Orange lines indicate marker positions.

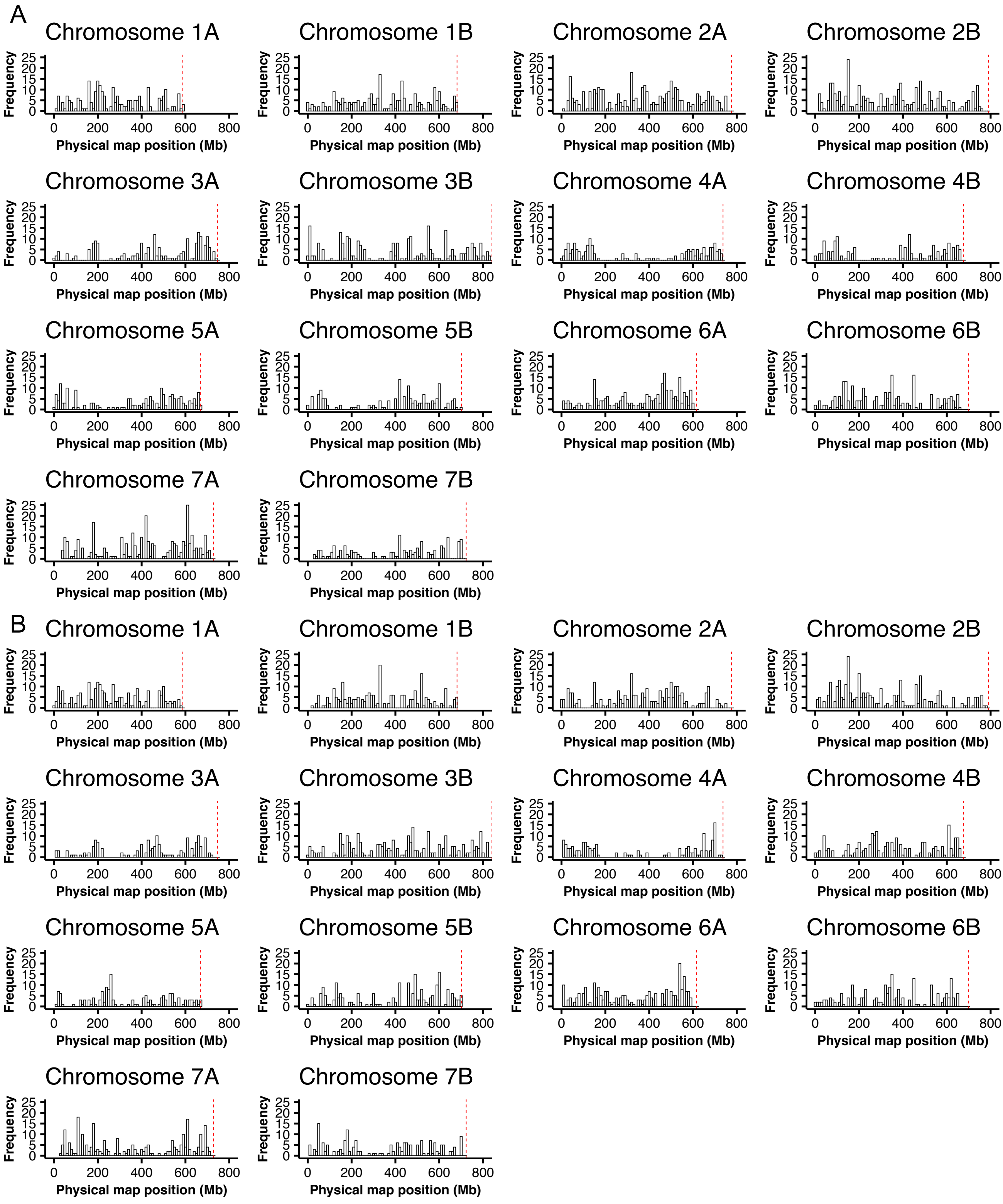

Supplementary Figure S10. Distribution of the number of SNPs/indels per 10 Mb in each chromosome. a) Distribution of markers used for linkage mapping of F2 population every 10 Mb on the genome b) Distribution of markers used for linkage mapping of $F_{6:7}$ RILs every 10 Mb on the genome. Dotted red lines indicate the end of chromosomes.

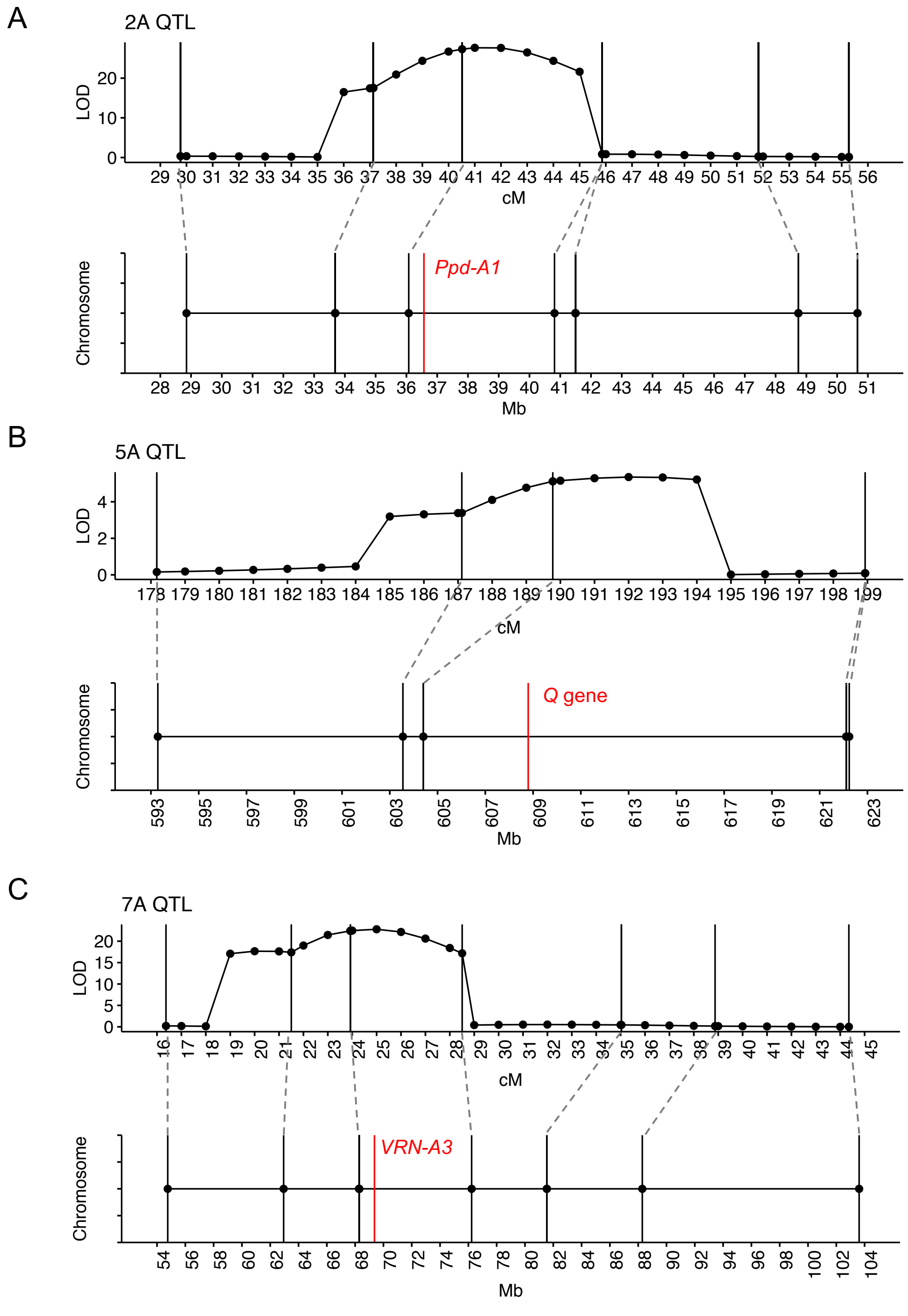

Supplementary Figure S11. Correspondence between physical map and linkage map positions of markers on chromosome 2A, 5A, and 7A in the $F_{6:7}$ RILs and 7A QTL of F_2 population with LOD score and relationship between each marker on the physical map and the position of *VRN-A3*, *Ppd-A1*, and *Q* gene.
a) 2A QTL of $F_{6:7}$ RILs. b) 5A QTL of $F_{6:7}$ RILs. c) c) 7A QTL of $F_{6:7}$ RILs. d) 7A QTL of the F_2 population.
Black lines indicate marker positions. Red lines indicate the physical positions of candidate genes for each QTLs

D

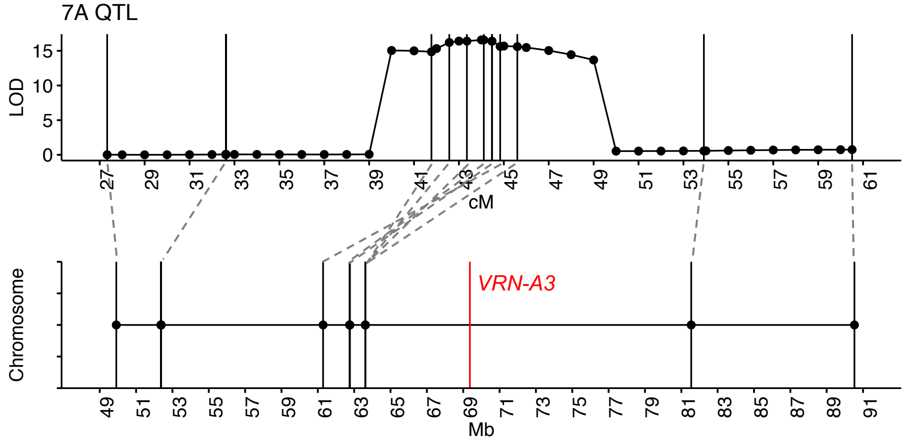

Supplementary Figure S11. Correspondence between physical map and linkage map positions of markers on chromosome 2A, 5A, and 7A in the $F_{6:7}$ RILs and 7A QTL of F_2 population with LOD score and relationship between each marker on the physical map and the position of *VRN-A3*, *Ppd-A1*, and *Q* gene.
a) 2A QTL of $F_{6:7}$ RILs. b) 5A QTL of $F_{6:7}$ RILs. c) c) 7A QTL of $F_{6:7}$ RILs. d) 7A QTL of the F_2 population.
Black lines indicate marker positions. Red lines indicate the physical positions of candidate genes for each QTLs

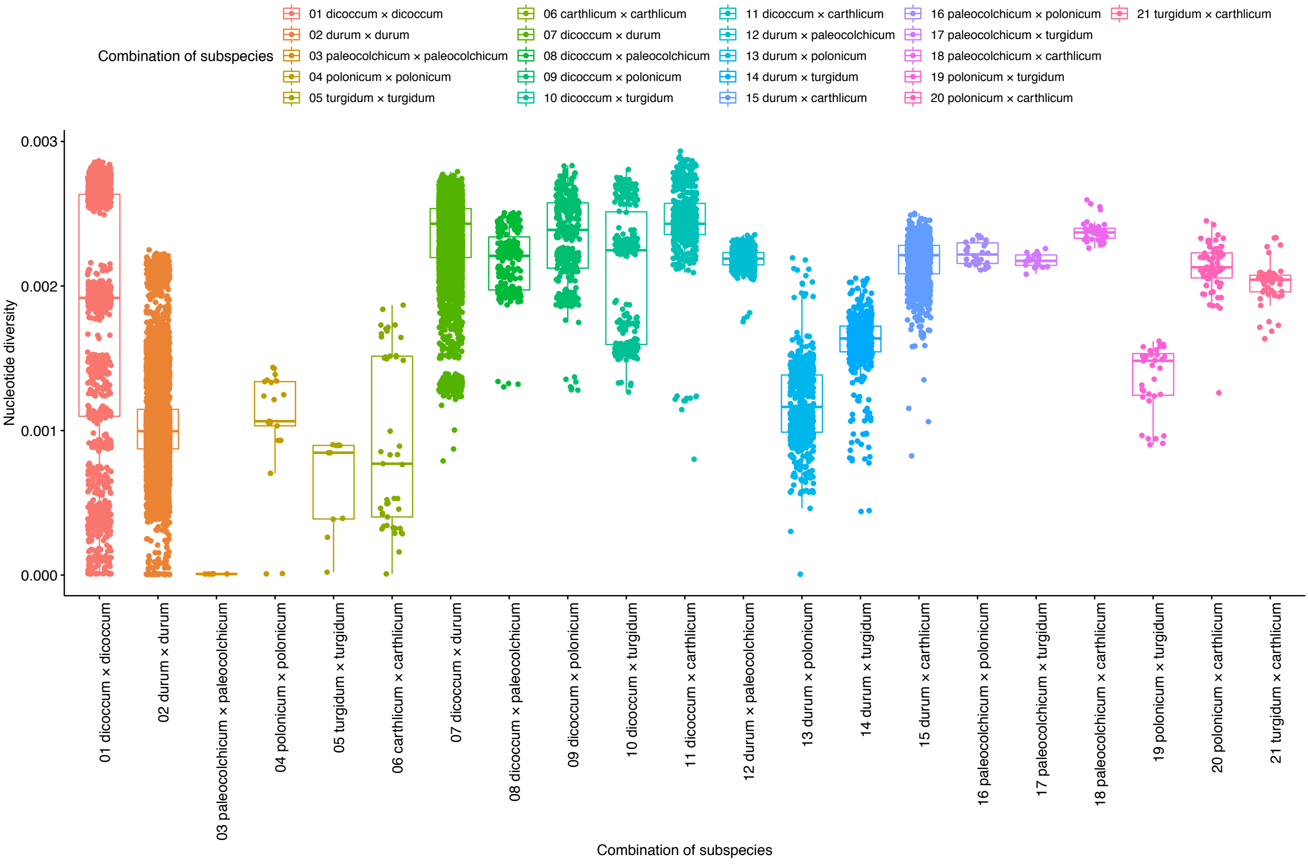

Supplementary Figure S12. Distribution of nucleotide diversity of all combinations of two accessions in TWC. The box-plots divided by inter- and intrasubspecies, respectively.

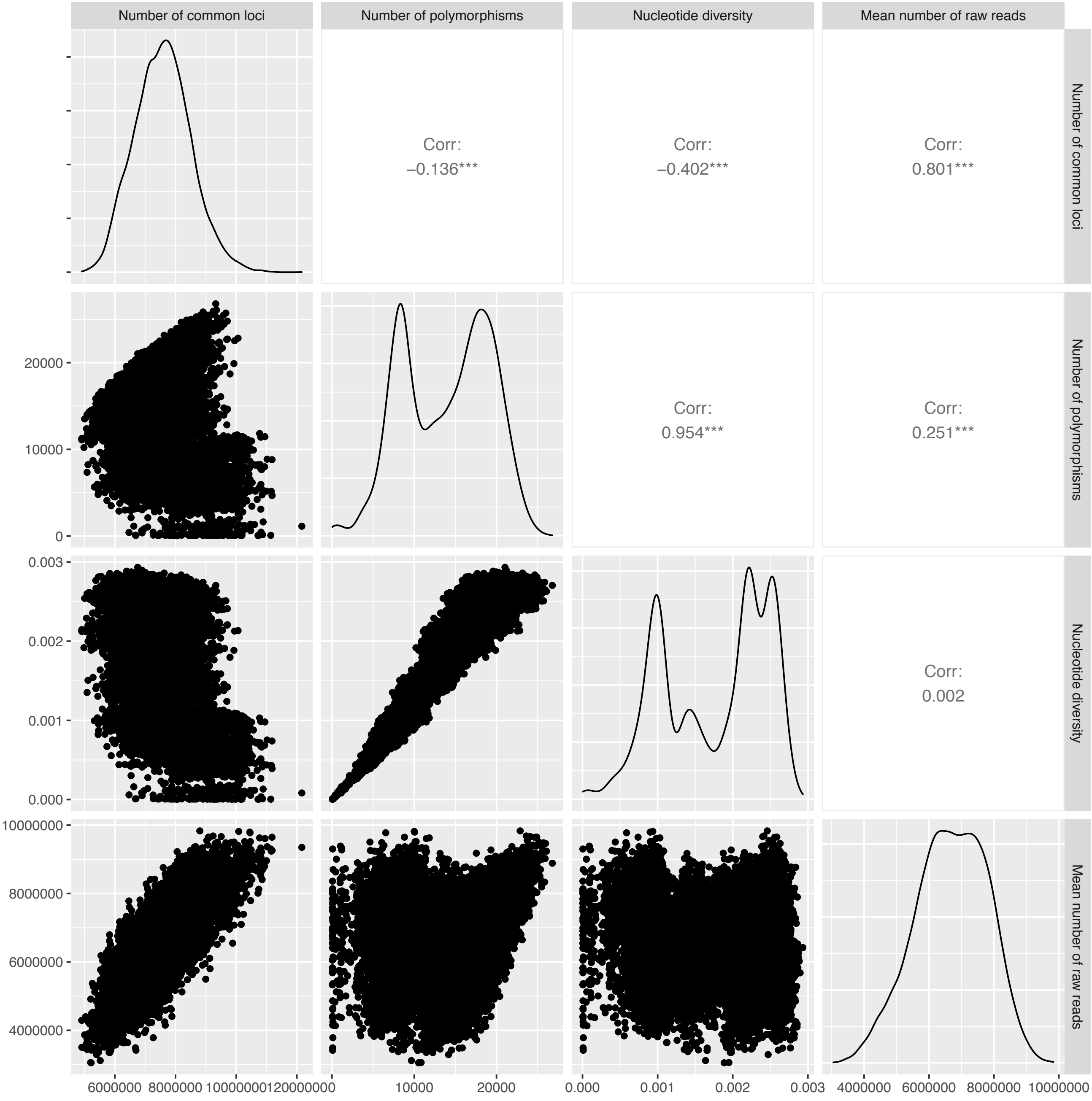

Supplementary Figure S13. Correlation analysis for identifying factors determining the number of polymorphisms between two accessions in TWC. "Number of common loci" is the total number of nucleotides between the two TWC lines that have a coverage depth greater than 5 in both accessions. " Number of polymorphisms " is the number of SNP/indels with coverage depth greater than 5 between the two TWC accessions. “Mean number of raw reads" is the average number of reads between the two TWC accessions. "Number of common loci”, " Number of polymorphisms " , Mean number of raw reads" , and nucleotide diversity were calculated in all combination of two accessions in TWC. "Corr" means the correlation coefficient.

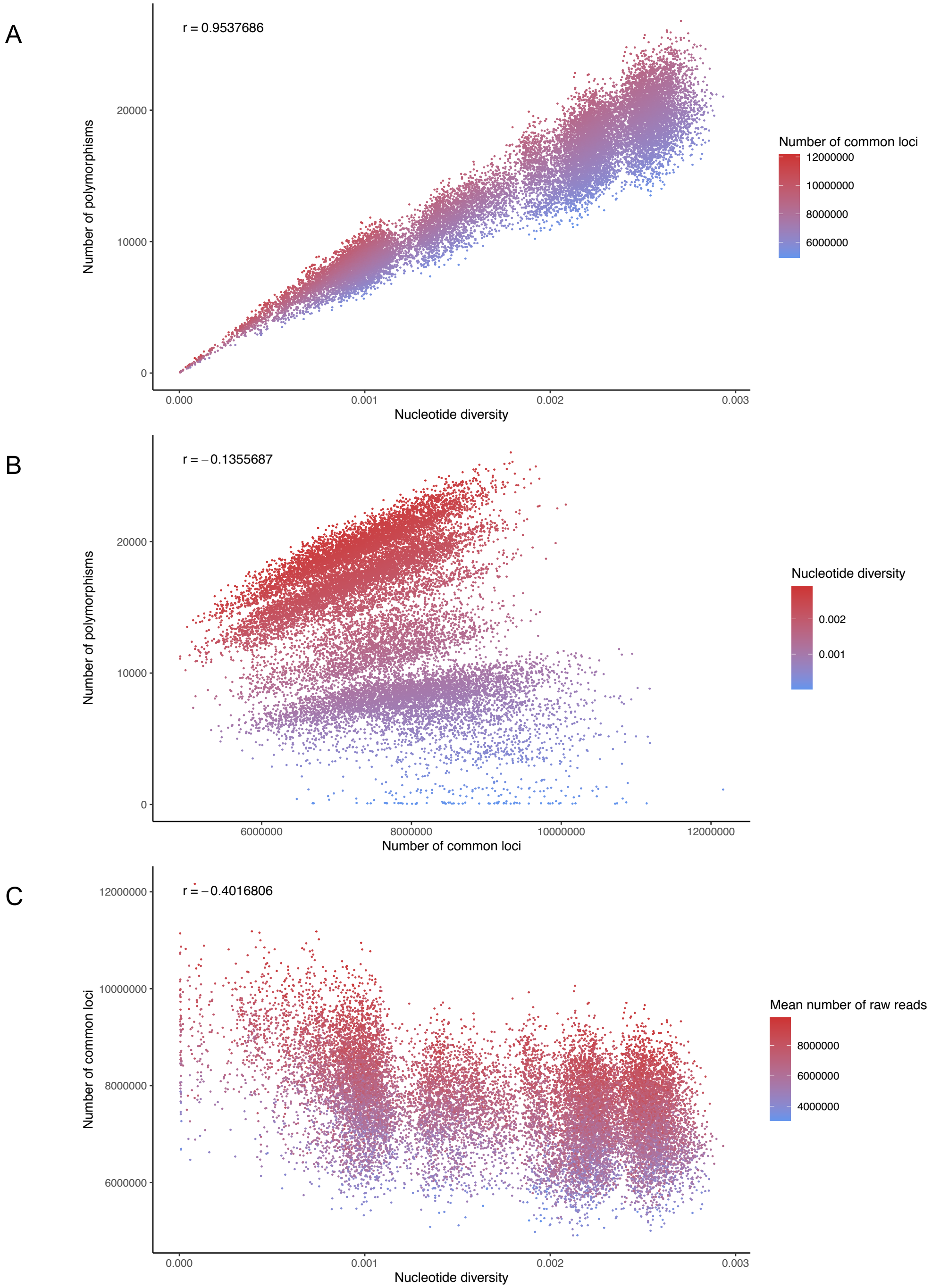

Supplementary Figure S14. Analysis of factors related with the number of polymorphisms between two accessions in TWC. “Number of polymorphisms” means the number of polymorphisms (SNP/indels) with a coverage depth of 5 or more between two TWC accessions. “Nucleotide diversity” means ratio of number of SNPs with a coverage depth of 5 or more to the number of bases sequenced with a coverage depth of 5 or more for both accessions. “Number of common loci” means the number of bases sequenced with a coverage depth of 5 or more in both accessions. “Mean number of raw reads” means average data volume of number of raw reads between two accessions in TWC. "Number of common loci”, " Number of polymorphisms " , Mean number of raw reads" , and nucleotide diversity were calculated in all combination of two accessions in TWC The red color scale indicates that the number is larger, and the blue color scale indicates that the number is smaller. a) Scatter plots of number of polymorphisms and nucleotide diversity. Color scale indicates number of common loci. b) Scatter plots of number of SNPs and common loci between two accessions. Color scale indicates nucleotide diversity. c) Scatter plots of number of common loci and nucleotide diversity. Color scale indicates mean number of raw reads.

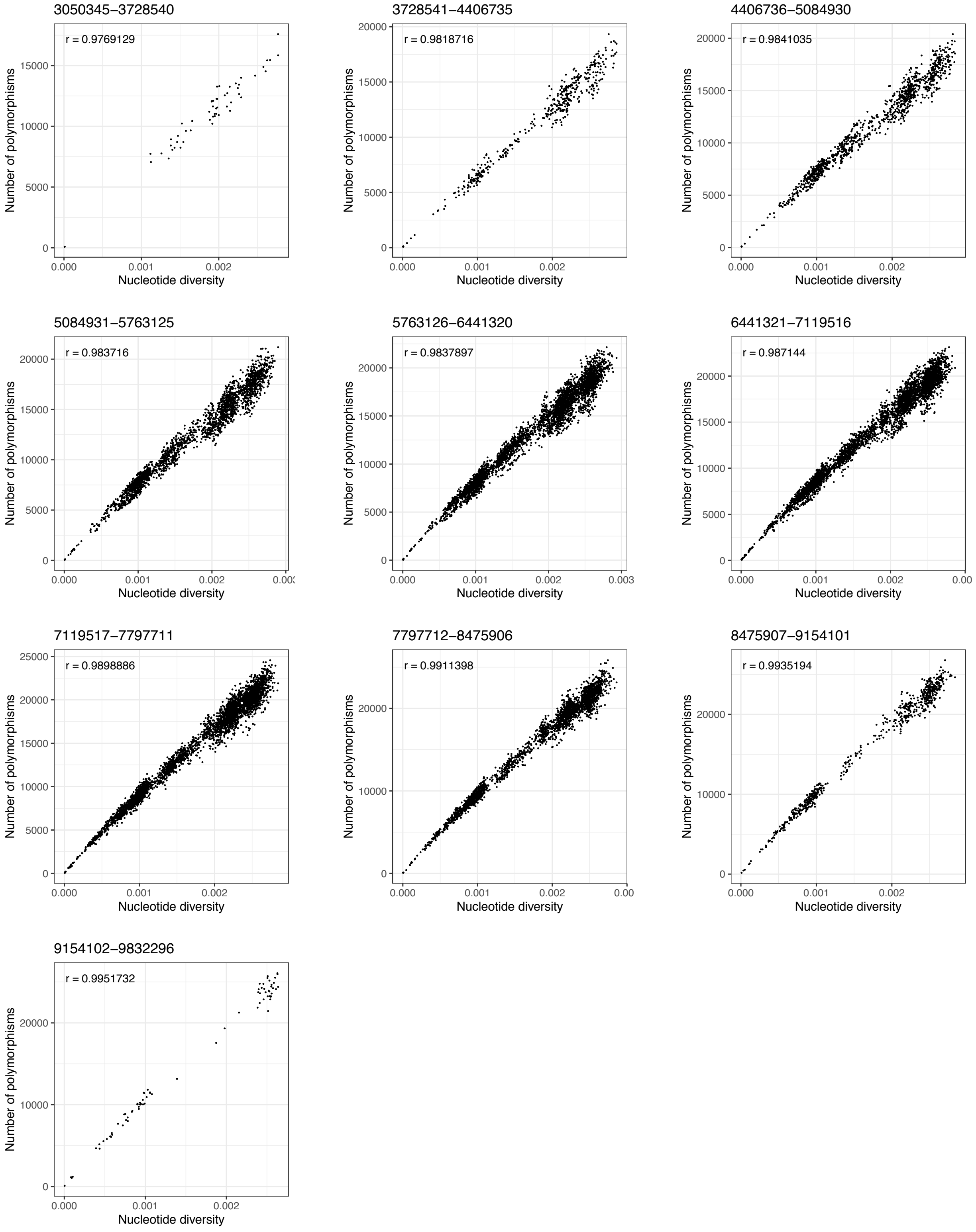

Supplementary Figure S15. Relationship between the number of polymorphisms and nucleotide diversity for each mean number of raw reads in TWC. Mean number of raw reads is the average number of raw reads between two accessions in TWC. The data volume ranges are shown at the top of each scatter plot. “r” denotes correlation coefficient between number of polymorphisms and nucleotide diversity.
